# Pathogenic variants in glutamyl-tRNA^Gln^ amidotransferase subunits cause a lethal mitochondrial cardiomyopathy disorder

**DOI:** 10.1038/s41467-018-06250-w

**Published:** 2018-10-03

**Authors:** Marisa W. Friederich, Sharita Timal, Christopher A. Powell, Cristina Dallabona, Alina Kurolap, Sara Palacios-Zambrano, Drago Bratkovic, Terry G. J. Derks, David Bick, Katelijne Bouman, Kathryn C. Chatfield, Nadine Damouny-Naoum, Megan K. Dishop, Tzipora C. Falik-Zaccai, Fuad Fares, Ayalla Fedida, Ileana Ferrero, Renata C. Gallagher, Rafael Garesse, Micol Gilberti, Cristina González, Katherine Gowan, Clair Habib, Rebecca K. Halligan, Limor Kalfon, Kaz Knight, Dirk Lefeber, Laura Mamblona, Hanna Mandel, Adi Mory, John Ottoson, Tamar Paperna, Ger J. M. Pruijn, Pedro F. Rebelo-Guiomar, Ann Saada, Bruno Sainz, Hayley Salvemini, Mirthe H. Schoots, Jan A. Smeitink, Maciej J. Szukszto, Hendrik J. ter Horst, Frans van den Brandt, Francjan J. van Spronsen, Joris A. Veltman, Eric Wartchow, Liesbeth T. Wintjes, Yaniv Zohar, Miguel A. Fernández-Moreno, Hagit N. Baris, Claudia Donnini, Michal Minczuk, Richard J. Rodenburg, Johan L. K. Van Hove

**Affiliations:** 1 Section of Clinical Genetics and Metabolism, Department of Pediatrics, University of Colorado, Aurora, 80045 CO USA; 2 Radboud Center for Mitochondrial Medicine, Translational Metabolic Laboratory, Department of Pediatrics, Radboud University Medical Center, Nijmegen, 6500 HB The Netherlands; 3 Department of Human Genetics, Radboud Institute for Molecular Life Sciences and Donders Centre for Neuroscience, Radboud University Medical Center, Nijmegen, 6500 HB The Netherlands; 4 Medical Research Council, Mitochondrial Biology Unit, University of Cambridge, Cambridge, CB2 OXY United Kingdom; 5 Department of Chemistry, Life Sciences and Environmental Sustainability, University of Parma, Parma, 43124 Italy; 6 The Genetics Institute, Rambam Health Care Campus, Haifa, 3109601 Israel; 7 The Ruth and Bruce Rappaport Faculty of Medicine, Technion – Israel Institute of Technology, Haifa, 3109601 Israel; 8 Departamento de Bioquímica, Instituto de Investigaciones Biomédicas “Alberto Sols” UAM-CSIC and Centro de Investigación Biomédica en Red en Enfermedades Raras (CIBERER). Facultad de Medicina, Universidad Autónoma de Madrid, Madrid, 28029 Spain; 9 Instituto de Investigación Sanitaria Hospital 12 de Octubre (imas12), Madrid, 28041 Spain; 10 SA Pathology, Women and Children’s Hospital Adelaide, Adelaide, 5006 Australia; 11 Division of Metabolic Diseases, Beatrix Children’s Hospital, University Medical Center Groningen, University of Groningen, Groningen, 9700 RB The Netherlands; 12 HudsonAlpha Institute for Biotechnology, Huntsville, AL 35806 USA; 13 Department of Genetics, University Medical Center of Groningen, University of Groningen, Groningen, 9700 RB The Netherlands; 14 Department of Pediatrics, Section of Pediatric Cardiology, Children’s Hospital Colorado, University of Colorado, Aurora, CO 80045 USA; 15 Department of Human Biology, Faculty of Natural Sciences, University of Haifa, Haifa, 3498838 Israel; 16 Department of Pathology, Children’s Hospital Colorado, University of Colorado, Aurora, 80045 CO USA; 17 Institute of Human Genetics, Galilee Medical Center, Nahariya, 22100 Israel; 18 The Azrieli Faculty of Medicine in the Galilee, Bar Ilan University, Safed, 1311502 Israel; 19 Department of Biochemistry and Molecular Genetics, University of Colorado, Aurora, CO 80045 USA; 20 Department of Pediatrics, Bnai Zion Medical Center, Haifa, 3339419 Israel; 21 Department of Human Genetics, Radboud Institute for Molecular Life Sciences and Donders Centre for Neuroscience, Radboud University Medical Center, Nijmegen, 6500 HB The Netherlands; 22 Metabolic Unit, Rambam Health Care Campus, Haifa, 3109601 Israel; 23 Department of Biomolecular Chemistry, Institute for Molecules and Materials, Radboud University, Nijmegen, 6500 HB The Netherlands; 24 Graduate Program in Areas of Basic and Applied Biology (GABBA), University of Porto, Porto, 4200-135 Portugal; 25 Monique and Jacques Roboh Department of Genetic Research and the Department of Genetic and Metabolic Diseases, Hadassah-Hebrew University Medical Center, Jerusalem, 91120 Israel; 26 Enfermedades Crónicas y Cáncer Area, Instituto Ramón y Cajal de Investigación Sanitaria (IRYCIS), Madrid, 28034 Spain; 27 Department of Pathology and Medical Biology, University Medical Center Groningen, University of Groningen, 9700 RB Groningen, The Netherlands; 28 Division of Neonatology, Beatrix Children’s Hospital, University Medical Center Groningen, University of Groningen, Groningen, 9700 RB The Netherlands; 29 Institute of Genetic Medicine, Newcastle University, Newcastle, NE1 3BZ United Kingdom; 30 Institute of Pathology, Rambam Health Care Campus, 3109601 Haifa, Israel

## Abstract

Mitochondrial protein synthesis requires charging mt-tRNAs with their cognate amino acids by mitochondrial aminoacyl-tRNA synthetases, with the exception of glutaminyl mt-tRNA (mt-tRNA^Gln^). mt-tRNA^Gln^ is indirectly charged by a transamidation reaction involving the GatCAB aminoacyl-tRNA amidotransferase complex. Defects involving the mitochondrial protein synthesis machinery cause a broad spectrum of disorders, with often fatal outcome. Here, we describe nine patients from five families with genetic defects in a GatCAB complex subunit, including *QRSL1*, *GATB*, and *GATC*, each showing a lethal metabolic cardiomyopathy syndrome. Functional studies reveal combined respiratory chain enzyme deficiencies and mitochondrial dysfunction. Aminoacylation of mt-tRNA^Gln^ and mitochondrial protein translation are deficient in patients’ fibroblasts cultured in the absence of glutamine but restore in high glutamine. Lentiviral rescue experiments and modeling in *S. cerevisiae* homologs confirm pathogenicity. Our study completes a decade of investigations on mitochondrial aminoacylation disorders, starting with *DARS2* and ending with the GatCAB complex.

## Abstract (web summary)

Mitochondrial protein synthesis requires charging a mitochondrial tRNA with its amino acid. Here, the authors describe pathogenic variants in the GatCAB protein complex genes required for the generation of glutaminyl-mt-tRNA^Gln^, that impairs mitochondrial translation and presents with cardiomyopathy.

## Introduction

**Fig. 1:**
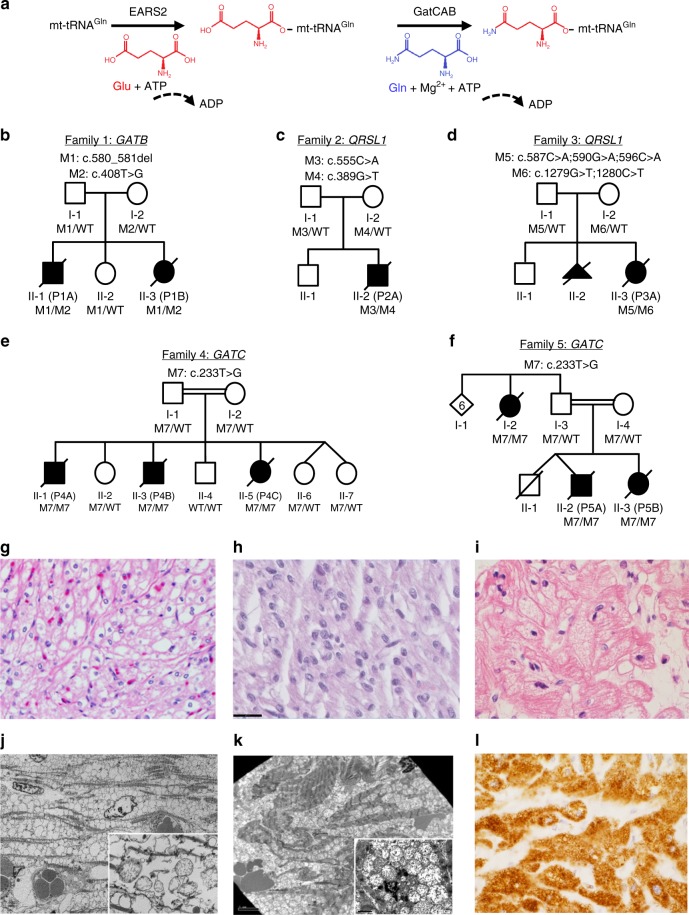
Pathogenic variants in GatCAB subunits lead to a lethal metabolic cardiomyopathy. **a** Mitochondrial-tRNA^Gln^ is charged with glutamine in a two-step reaction. **b**–**f** Pedigrees of the families with pathogenic variants in *QRSL1*, *GATB*, or *GATC* as identified in the patients. **g**–**l** Pathology of patients' heart tissues. **g**–**i** Light microscopy on hematoxylin-eosin staining showed pericardial clearing in patients **g** P3A (GatA, magnification 100X), **h** P1A (GatB, magnification 100X (scale bar 20 μm), and **i** P4A (GatC, magnification 100X). **j**, **k** Electron microscopy of the heart showed extensive mitochondrial proliferation displacing the contractile elements in patients **j** P3A (GatA, magnification 2700X, insert magnification 27,000X) and **k** P4A *(*GatC, magnification 2500X (scale bar 5 μm), insert magnification 12,000X (scale bar 2 μm). **l** SDHB immunostaining in patient P4A (GatC) showed massive increase in mitochondria in the heart (magnification 100X)

Mitochondrial disorders are highly heterogeneous due to their complex biochemistry and genetics. The oxidative phosphorylation system (OXPHOS) is essential for proper ATP production and organism function. It consists of 98 proteins distributed across five multi-subunit complexes (I–V) encoded by both the nuclear and mitochondrial genomes. The 13 mtDNA-encoded subunits belong to complexes I, III, IV, and V, and all the subunits belonging to complex II are encoded by nuclear DNA. The nuclear encoded mitochondrial genes are transcribed in the nucleus, their mRNAs are translated in the cytosol and proteins are imported to the mitochondria through a complex import machinery. OXPHOS system biogenesis is therefore the result of precise and coordinated cytosolic and mitochondrial translational processes, the end result of which is to obtain the appropriate stoichiometric levels of OXPHOS subunits to assemble into functional complexes. The mitochondrial DNA-encoded subunits are transcribed and then translated on the mitochondrial ribosomal machinery prior to assembly into the complexes. Precise mRNA-to-protein translation by the mitochondrial protein synthesis machinery^1^ is necessary for OXPHOS assembly, and requires that each tRNA is paired with the correct amino acid to allow that codon-anticodon pairing results in proper protein formation^2^. In the mitochondria, this process is mediated by the mitochondrial aminoacyl-tRNA synthetases (ARS2s), which are encoded by nuclear genes. Of these, 17 ARS2 are unique to the mitochondria, while *GARS* (Glycyl-tRNA synthetase) and *KARS* (Lysyl-tRNA synthetase), are encoded by the same loci as the cytoplasmic enzymes, with the mitochondrial isoforms being generated by alternative translation initiation (*GARS*)^3^ or alternative splicing (*KARS*)^4^. By exception, glutaminyl mt-tRNA (mt-tRNA^Gln^) is aminoacylated by an indirect pathway^5^, in which it is first charged with glutamic acid (Glu) by mitochondrial glutamyl-tRNA synthetase (EARS2), after which the Glu-mt-tRNA^Gln^ is transamidated into Gln-mt-tRNA^Gln^, using free glutamine as an amide donor (Fig. 1a)^5^. This latter conversion is performed by GatCAB, the glutamyl-tRNA^Gln^ amidotransferase protein complex, that consists of three subunits: GatA encoded by *QRSL1*, GatB encoded by *GATB*, and GatC encoded by *GATC*^5,6^.

Over the past decade, pathogenic variants in all ARS2 genes and in *QRSL1*^7,8^ have been associated with a variety of metabolic phenotypes^9^. In this report, we provide evidence of mitochondrial function defects caused by GatCAB pathogenic variants, including gene defects in *GATB* and *GATC*. Patients present with metabolic cardiomyopathy and have defective Gln-mt-tRNA^Gln^ acylation, resulting in reduced mitochondrial protein translation with an influence of the amount of glutamine available. Thus, the account of mitochondrial aminoacylation defects in human disease, that started with the discovery of pathogenic mutations in *DARS2*^10^ in 2007, is now completed by the discovery of defects in *GATB* and *GATC*.

## Results and Discussion

### Clinical presentation

We studied five families (Fig. 1b–f) with infants predominantly exhibiting severe cardiomyopathy and fatal lactic acidosis, which raised suspicion of mitochondrial disease. The onset of symptoms was either prenatal (families 1 and 3) or infantile at 2–5 months (families 2, 4, and 5), and no child survived beyond 6.5 months. Anemia, which is a rare symptom in mitochondrial disorders^11–13^, was frequently present, ranging in severity from mild to severe prenatal anemia requiring intrauterine transfusions. The bone marrow biopsy did not show ringed sideroblasts in any of the subjects. Additional features present in several subjects include liver dysfunction, mildly elevated creatine kinase levels, and hydropic features including pericardial effusion. Postmortem biopsies revealed cardiomyocytes with massive mitochondrial proliferation, as observed on electron microscopy and on histology represented by rarefactions in the cytoplasm staining (Fig. 1g–l). Biochemical investigations showed abnormalities of the mitochondrial respiratory chain enzymes (Supplementary Table 1). The complete clinical presentations of the patients are summarized in Table 1, and the full clinical reports are available in Supplementary Note 1. A similar presentation of early lethal cardiomyopathy was noted in the few previously reported *QRSL1* cases^7,8^.

**Table 1:** Symptoms of patients with deficient GatCAB complex

		P1A	P1B	P2A	P3A	P4A	P4B	P4C	P5A	P5B
**Gender**		M	F	M	F	M	M	F	M	F
**Age at onset**		Prenatal 34 weeks	Prenatal 30 weeks	3 months	Prenatal 13 weeks	3 months	2 months	3 months	5.5 months	3 months
**Age at death**		2 days	1 day	3 months	1 day	3 months	2 months	3.5 months	6.5 months	6.5 months
**Affected gene**		*GATB*	*GATB*	*QRSL1*	*QRSL1*	*GATC*	*GATC*	*GATC*	*GATC*	*GATC*
**Prenatal**	**Frequency**									
Hydrops	3/9	√	√	–	√	–	–	–	–	–
IUGR	2/9	√	√	–	–	–	–	–	–	–
**Postnatal**										
Prematurity	2/9	√	–	–	√	–	–	–	–	–
Cardiomyopathy	9/9	√	√	√	√	√	√	√	√	√
Lactic acidosis	9/9	√	√	√	√	√	√	√	√	√
Anemia	7/7	√	√	√	√	√	NA	NA	√	√
Hepatic dysfunction	5/9	–	–	√	–	√	√	√	√	–
Elevated CK	5/9	–	–	–	√	√	√	√	√	–
Hypoglycemia	2/9	–	√	√	–	–	–	–	–	–
Hearing loss	1/1	NA	NA	√	NA	NA	NA	NA	NA	NA
Low cortisol	1/2	–	NA	NA	√	NA	NA	NA	NA	NA

Frequency of symptoms identified in the patients with defects in the GatCAB complex

*√* symptom present, *-* symptom not present, *CK* creatine kinase, *IUGR* intrauterine growth restriction, *NA* data not available

### Genetics

Whole exome sequencing (WES) analysis in these families uncovered rare pathogenic variants in the three genes encoding the GatCAB complex subunits, in-line with autosomal recessive inheritance (Fig. 1b–f, Supplementary Table 2 and Supplementary Fig. 1). In family 1, compound heterozygous variants were identified in *GATB*: paternal c.580_581del; p.Ser194Trpfs*15 and maternal c.408T>G; p.Phe136Leu. In family 2, we identified compound heterozygous variants in *QRSL1*: paternal c.555C>A; p.Tyr185* and maternal c.398G>T; p.Gly133Val, which has been previously described^7^. In family 3, WES revealed compound heterozygous variants in *QRSL1*: paternal c.[587C>A;590G>A;596C>A]; p.[Thr196Asn;Arg197Lys;Pro199His] and maternal c.[1279G>T;1280C>T]; p.Ala427Leu. In families 4 and 5, which are reportedly not related but reside in adjacent villages, we identified a homozygous missense variant in *GATC*: c.233T>G; p.Met78Arg. Segregation analysis confirmed co-segregation among available affected and healthy siblings and parents in all families. All missense variants were predicted to be deleterious by the bioinformatics programs PolyPhen2 and MutationTaster, and most by SIFT (Supplementary Table 2); only Gly133Val was predicted as tolerated by SIFT, yet this variant has been previously shown as functionally damaging^7^.

Due to their essential role in protein synthesis, residual enzyme activity characterizes disorders of tRNA synthetases, and complete loss-of-function is thought to be embryonically lethal^9,14,15^. Therefore, a pathogenic variant in tRNA-charging genes should be damaging enough to prevent normal respiratory chain enzyme activity, whereas, mild enough to maintain residual respiratory chain enzyme activity required for viability^9,14^. All patients reported herein have at least one missense allele that may account for residual GatCAB activity. In addition, we observed a possible relation between clinical severity and the degree of amino acid conservation, i.e., the most severely affected families 1 and 3 with prenatal onset and neonatal demise had a missense variant involving highly conserved amino acids, whereas families 2, 4, and 5 with infantile onset had missense variants that affected moderately conserved amino acids (Supplementary Fig. 1). In many biochemical genetic conditions, the phenotypic severity relates to the amount of residual enzyme activity, which could be due to the effect of missense mutations, or, more rarely, leaky splicing defects, or penultimate 3’stop mutations. A leaky splice site mutation in *QRSL1* was previously reported^8^. In the patients presented here, frameshift and null mutations are likely to result in complete loss of protein expression. Thus, we postulate that the phenotypic variability relates to the level of residual activity of each missense mutation, with the severity of their functional effect reflected in the amino acid conservation and the effect on the protein structure and function in the modeling below, with the most stringently conserved amino acids related to the earlier presentation.

### Molecular modeling of protein structure and function

**Fig. 2:**
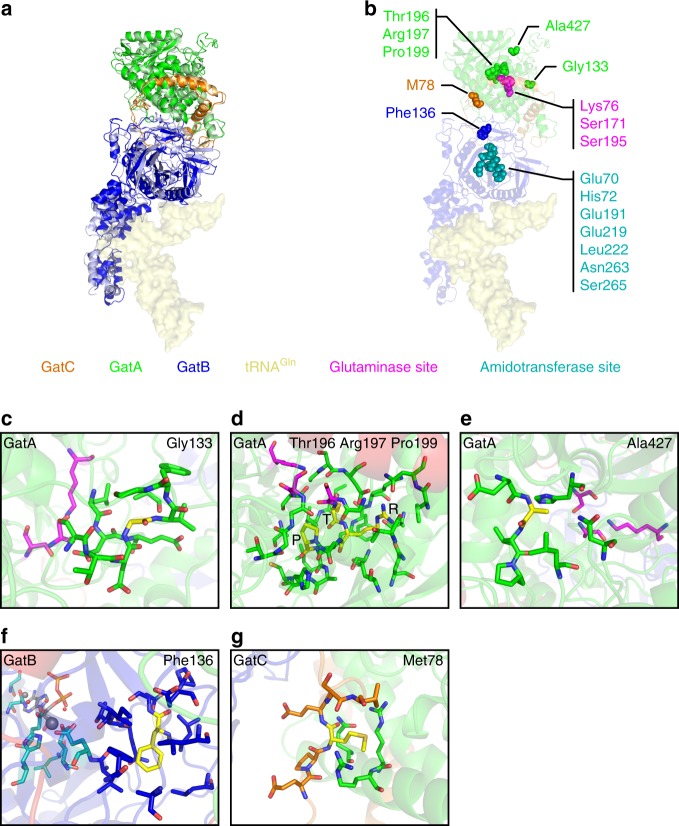
Modeling of the *Homo sapiens* GatCAB complex and mapping of variants. **a** Representation of the bacterial GatCAB (light colors) with bound tRNA^Gln^ (light yellow surface) and the superimposed modeled human complex (GatA in green, GatB in blue, and GatC in orange). **b** Mapping of residues involved in catalysis at glutaminase site in GatA (magenta) and at the amidotransferase site in GatB (teal), and residues found to be mutated in patients, to the structure of the human GatCAB model. **c**–**g** Inspection of the microenvironment of mutated residues (yellow) in the human GatCAB model. Color-coding for the individual subunits of the GatCAB complex as in **a** and **b**. In each case, the mutated amino acid is shown in yellow, GatA residues in green, GatB residues in blue, glutaminase side in magenta; nitrogen is shown in dark blue and oxygen in red

In the bacterial GatCAB complex, GatA and GatB possess the catalytic amidase and kinase functions, respectively, while GatC serves as a stabilizing linker between them^16^. To discern the effects that the mutations have on the structure and function of this enzyme, the variants identified in the patients were modeled in the human GatCAB complex (Fig. 2a, b). In GatB, Phe136 mutated in P1A is located in a hydrophobic region, in close proximity to the catalytic residues of the amidotransferase site and to the GatA–GatB interface (Fig. 2f). The Phe136Leu mutation preserves the hydrophobic character of the region, but the reduced side chain size could reposition residues forming this hydrophobic core with potential long-range impact, and the absence of the phenyl-ring could weaken the π–π interaction with GatB Phe82. Owing to large-scale effects and the proximity of the affected residue to the amidotransferase site, the Phe136Leu mutation can lead to reduced catalytic activity.

In GatA, Gly133 mutated in P2A is located in a loop close to the glutaminase center and is crucial for this region’s conformation by promoting a β-turn structure though backbone interaction with Ser130, and in the absence of a sidechain allowing greater flexibility to the loop (Fig. 2c). The Gly133Ala mutation introduces an aliphatic sidechain, which would contrapose these contributions to the structure indirectly perturbing the conformation of its catalytic site. This predicted deleterious effect explains the previously published pathogenicity^7^.

The residues Thr196, Arg197, and Pro199 mutated in P3A are buried within GatA close to its glutaminase center, and their mutations likely affect structure and function (Fig. 2d). Especially critical is Thr196, which directly contacts Ser195, the catalytic residue, and its side chain is surrounded by the backbone of residues composing the β-sheet of GatA and by the side chain of Leu233, to which its ramified aliphatic portion establishes hydrophobic interactions. The alteration in Thr196Asn to a bulkier asparagine could not only sterically perturb the β-sheet structure, but also shift the position of the connecting catalytic Ser195 affecting catalysis. Residue Arg197 is located in a polar pocket where it interacts with carboxyl and carbonyl groups of surrounding aspartate, asparagine, and glutamine residues. Despite similar physicochemical properties of arginine and lysine, the guanidinium group of arginine is able to establish more simultaneous interactions than the ε-amino group of lysine, providing more stabilizing interactions; hence Arg197Lys may compromise the stability of its vicinity. The non-polar ring of Pro199 is exposed to the backbone of catalytic residues Ser195 and Ser171 responsible for glutamine intermediate formation and charge relay. The Pro199His mutation may impact the helix structure, and the larger size of the histidine side chain may cause steric disturbances, and may further establish new interactions with the backbone of neighboring residues of the glutaminase center, which displacement may impact catalysis.

Ala427 mutated in P3A is exposed on the surface of GatA, away from surfaces of interaction with the other subunits of the GatCAB complex, and in the Ala427Leu mutation both amino acids are hydrophobic, with only an increased side chain size with limited steric effect since this residue is located in a solvent-exposed surface, thus predicting limited impact likely with some residual activity present (Fig. 2e).

In GatC, Met78 mutated in families 4 and 5 is located at the interface of the GatC subunit with GatA where this electron-rich residue interacts with the aliphatic segment of two positively charged arginine residues (Fig. 2g). The Met78Arg mutation disrupts these interactions by electrostatic repulsion compromising the interaction between GatC and GatA.

### GatCAB complex levels

**Fig. 3:**
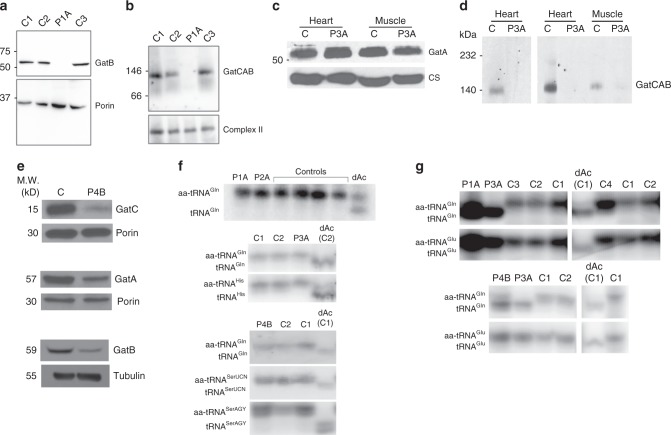
GatCAB protein expression levels and mitochondrial tRNA aminoacylation analysis. **a** Western blot after SDS-PAGE shows a near absence of GatB protein in the fibroblasts of patient P1A in contrast to three controls (C#1–3), with porin and tubulin as loading controls; **b** Western blot after BN-PAGE show the GatCAB protein complex as an approximately 120 kDa complex in controls, but is not detectable in P1A fibroblasts, with complex II used as loading control. **c** Western blot from both control (C) and patient P3A skeletal and heart muscle shows normal amount and size of the GatA protein, with citrate synthase (CS) as loading control. **d** The assembly of the GatCAB complex on native polyacrylamide gradient gel and western blot probed with anti-GatB antibody in heart and muscle tissues of both control (C) and patient P3A shows absence of the holocomplex at approximately 120 kDa in patient samples. **e** Western blot analysis of control and patient P4B fibroblasts showed a strong reduction in GatC protein, and also reduced levels of GatA and GatB protein, with porin as loading control. **f** Northern blot analysis of mitochondrial tRNA aminoacylation in total RNA samples from patients P1A and P2A, and control human fibroblasts show similar glutamine charging of mt-tRNA^Gln^ when cells were grown in standard glutamine-containing medium, with similar results for fibroblasts from P3A and P4B. **g** After 3 days in culture medium without glutamine, fibroblasts from P1A, P3A, and P4B show decreased glutamine charging of mt-tRNA^Gln^ compared to control fibroblasts. “dAc” indicates deacylated control sample

The steady state protein levels of the GatCAB subunits are variably affected by the mutations as determined through western blot analysis. Fibroblasts from subject P1A with mutations in *GATB*, showed strongly reduced levels of the GatB protein in comparison to controls (Fig. 3a). The GatCAB holocomplex is approximately 120 kDa in control fibroblasts by blue native polyacrylamide gel electrophoresis (BN-PAGE) analysis, compatible with a complex consisting of one copy of each component, but in affected subjects’ fibroblasts this complex was not detected (Fig. 3b). In subject P3A with mutations in *QRSL1*, there is no effect on protein expression of the GatA protein between affected subject and controls in heart and skeletal muscle (Fig. 3c), yet the holocomplex at 120 kDa was consistently absent in the affected subject in muscle and heart tissues, but normal in cultured skin fibroblasts, which is compatible with the normal respiratory chain enzymes in these cells (Fig. 3d). In subject P4B with mutations in *GATC*, the steady state level of GatC in fibroblasts was decreased to 20% of controls (Fig. 3e), and the GatA and GatB protein levels were equally substantially decreased (Fig. 3e), probably due to the instability of the individual subunits when they are not incorporated in the GatCAB trimer. To exclude a genetic regulatory effect on the expression of *QRSL1*, *GATB*, or *GATC* provoked by the reduction of the GatCAB subunits in patient fibroblasts, we analyzed their mRNA levels in exponentially growing fibroblasts from patients P3A and P4B and found no significant changes in the transcriptional rate of the three genes compared to controls (Supplementary Fig. 2).

### A defect in mitochondrial translation

**Fig. 4:**
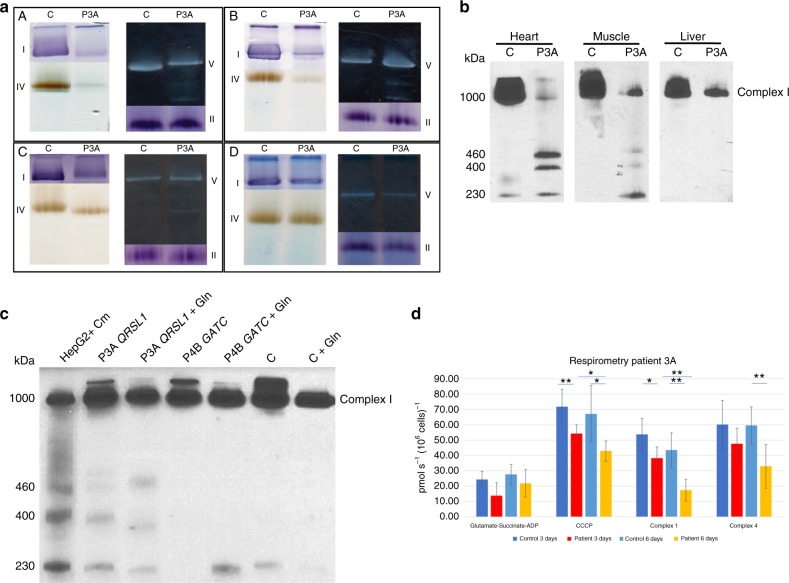
Mitochondrial function studies. **a** Blue native polyacrylamide gel electrophoresis with in-gel activity staining of solubilized respiratory chain enzyme complexes from a mitochondrial membrane pellet of heart muscle (**A**), skeletal muscle (**B**), liver (**C**), and fibroblasts (**D**) show decreased activities of complexes I and IV (except in fibroblasts) with additional low molecular weight bands of complex V in heart, muscle and to a lesser extent liver of patient P3A. **b** Analysis of the assembly of complex I in heart, skeletal muscle, and liver of patient P3A. The assembly of complex I was evaluated by separation on a native gel followed by western blotting and probing with an antibody against NDUFS2, a subunit which is present from the early stages of assembly. Abnormal subcomplexes of incompletely assembled complex I are visible in heart muscle, to a lesser extent in skeletal muscle, and absent in liver. **c** The assembly of complex I is shown for fibroblasts cultured in the presence (2 mM) or absence of added glutamine (+Gln) to the tissue culture media, with HepG2 cells cultured with chloramphenicol (HepG2+CM) showing typical subassembly intermediates, in comparison to control fibroblasts. **d** High-resolution respirometry using a SUIT protocol of fibroblasts from patient P3A is shown following withdrawal of glutamine for 3 and 6 days. Maximum coupled respiration using substrates pyruvate, glutamate, and succinate in the presence of ADP is not different, but after uncoupling with CCCP, the patient’s fibroblasts (*n* = 5) have significantly decreased rate compared to controls (five controls, average of triplicate analysis). Calculated complex I and complex IV rates are decreased in patient after 6 days withdrawal. Mean and standard deviation are shown, and differences evaluated by two-sided *t*-test. *p<0.05; **p<0.01

Analysis of the mitochondrial respiratory chain enzymes in all patients showed a combined deficiency with decreased activities of complexes I and IV, and low or borderline-low activity of complex III, that varied between the different tissues tested (Supplementary Table 1). In contrast to the fibroblasts of the GatA and GatC patients, clear respiratory chain enzyme deficiencies were observed in the fibroblasts of the GatB patients. BN-PAGE with in-gel activity staining of heart muscle, skeletal muscle, and liver samples from patient P3A (GatA) confirmed reduced activities of complexes I and IV with the presence of pathogenic lower molecular weight bands of complex V (Fig. 4a). In addition, observed subassemblies of complex I were present in heart and muscle (Fig. 4b), and were more prominent in fibroblasts when grown in medium without glutamine (Fig. 4c). Fibroblasts from patient P3A showed normal respiration compared to controls in culture medium containing glutamine, but after withdrawal of glutamine for three or six days, the CCCP-uncoupled maximum rate, and the calculated complex I and complex IV activities were significantly decreased (Fig. 4d). Taken together, these findings are consistent with a defect in mitochondrial translation with marked tissue specific differences most pronounced in heart, less in skeletal muscle, and near absent in fibroblasts, but exaggerated upon glutamine reduction.

Given the extensive tissue differences observed for the effects of the GatCAB complex genes in our subjects, the question arose whether another protein such as QARS could contribute to the aminoacylation of mt-tRNA^Gln^ in certain tissues such as fibroblasts. Certain previous publications and databases referred to cytoplasmic QARS as an ARS that operates in both mitochondrial and cytoplasmic compartments.^17–21^ To experimentally verify if QARS would localize in human mitochondria, we expressed QARS in fusion with green fluorescence protein (QARS-GFP) in human cells and studied its cellular localization using confocal microscopy. We did not detect any apparent co-localization of QARS-GFP with the mitochondrial protein TOM20 (Supplementary Fig. 3a, b). Furthermore, cellular fractionation experiments of human cells did not show any substantial enrichment of the endogenous QARS within the mitochondrial fraction (Supplementary Fig. 3c). The very low amount of QARS fractionating with mitochondria was sensitive to proteinase K treatment, confirming that that there is no detectable QARS within human mitochondria (Supplementary Fig. 3c). Therefore, mitochondria are exclusively dependent on the GatCAB complex for appropriate charging of mt-tRNA^Gln^ with glutamine. While aminoacylation of mt-tRNA^Gln^ appears to show only minor changes in comparison to controls when patient cells are grown under standard conditions with high concentrations of the GatCAB substrate glutamine in the culture medium (Fig. 3f), the aminoacylation in patient cells is strongly impaired in minimal essential culture medium that lacks the non-essential amino acid glutamine (Fig. 3g).

**Fig. 5:**
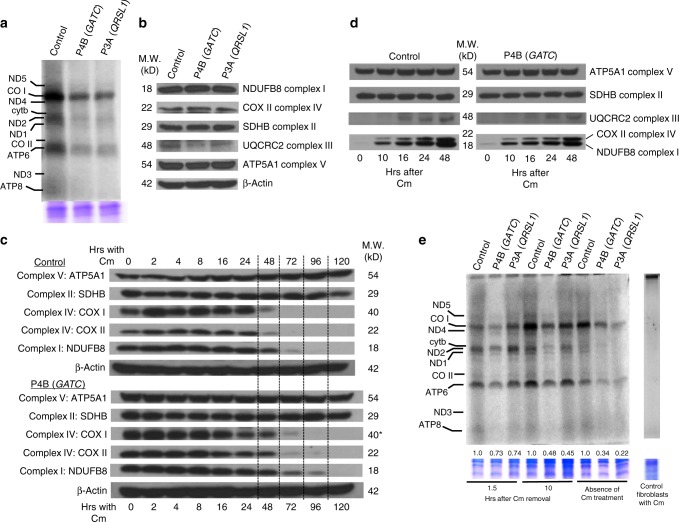
Synthesis of proteins of oxidative phosphorylation complexes in patient fibroblasts. OXPHOS protein synthesis in patient fibroblasts was analyzed by pulse labeling with [^35^S]methionine for 90 min in the presence of the cytosolic protein synthesis inhibitor emetine (**a**, **e**) using Coomassie staining as a loading control. Western blots show protein levels of NDUFB8 (complex I), UQCRC2 (complex III), ATP5A1 (complex V), COXI and COX II (complex IV) and SDHB (complex II) and β-actin (loading control) (**b**, **c**, **d**). NDUFB8 and UQCRC2 are labile if not incorporated into fully assembled complexes I and III, respectively, thus being an indirect indicator of the levels of mtDNA-encoded proteins within OXPHOS complexes. SDHB and ATP5A1 levels should not change (as it is shown) since both are encoded in the nucleus. In the absence of the complex V mtDNA-encoded proteins, ATP5A1 remains assembled and stable in the F1 subcomplex. **a** Decrease in newly synthesized peptides in GATA and GATC patient fibroblasts compared to control as determined by pulse labeling. Not all of the 13 mtDNA-encoded proteins can be seen. **b** Western Blot analysis showing that steady state levels of respiratory chain enzyme subunits are not reduced in patients P3A (*QRSL1*) and P4B (*GATC*) fibroblasts. Levels of NDUFB8 and UQCRC2 reflect the levels of mtDNA encoded proteins of complexes I and III, respectively. **c** Western blot analysis showing increased stability for NDUFB8, COXI, and COXII in patient P4B (*GATC*) fibroblasts after blocking mitochondrial protein synthesis with chloramphenicol (Cm) from 0 to 120 h. **d** Recovery of OXPHOS complexes containing mtDNA-encoded proteins in fibroblasts of patient P4B after release of mitochondrial protein synthesis blockade with chloramphenicol. **e** [^35^S]-methionine labeling of mitochondrial protein synthesis after a transient block with Cm. Chloramphenicol completely inhibits mitochondrial protein synthesis (right lane). Densitometric analysis was performed. Coomassie stained gels were used for protein loading normalization, images were analyzed with ImageJ, controls were set to 1.0, respective experimental samples were compared to the control, and fold changes are indicated below each lane. Average values for 2–3 experiments were pooled

The impact of the impairment in mt-tRNA^Gln^ charging on the mitochondrial protein synthesis capacity in the fibroblasts from subjects P3A and P4B was evaluated. Pulse labeling of mitochondrial proteins with [^35^S]-methionine in the presence of emetine, a cytoplasmic protein synthesis inhibitor, revealed a strong and generalized mtDNA-encoded protein synthesis defect after a 90-min pulse in P4B (*GATC*) and in P3A (*QRSL1*) patients’ fibroblasts (Fig. 5a). It is remarkable that this impairment of mitochondrial DNA-encoded protein synthesis correlates with no apparent changes in the steady state levels of representative OXPHOS proteins reflecting the assembled mitochondrial complexes (Fig. 5b). The nuclear DNA-encoded mitochondrial proteins NDUFB8 and UQCRC2 are unstable when not incorporated into their corresponding complexes, thus they are an accurate reflection of the levels of integral complexes I and III containing mtDNA-encoded proteins as previously described^22–26^. The possibility that the decrease of NDUFB8 and UQCRC2 levels is the result of a retrograde response seems improbable. RT-qPCR experiments showed no decrease in their mRNA levels in the mutant fibroblasts (Supplementary Fig. 4). Rather, NDUFB8 and COXVA mRNAs levels increase, compatible with a decrease in NDUFB8 protein levels as a consequence of its instability when it is not incorporated into its respective complex; in contrast, the mRNA levels of COXVA, which is part of a stable F1 subcomplex V, are unchanged. Further, the majority of retrograde responses to mitochondrial function defects, when they occur, results in the increase of compensatory protein levels and enzyme activities. Thus, the absence of changes in the steady state levels of COXII, NDUFB8, and UQCRC2 together with the reduced de novo synthesis of mitochondrial DNA-encoded proteins suggest a possible increase in the stability of the respiratory chain subunits in mutant cells. This was confirmed by incubation of P4B fibroblasts in the presence of chloramphenicol to arrest mitochondrial protein biosynthesis and harvesting these cells and analyzing them at different times for up to 120 h (Fig. 5c). The combined effect of an increase in stability as observed, together with residual GatCAB activity in fibroblasts, could explain the lack of substantive changes in steady state levels of complexes I, III, and IV.

We next analyzed the ability of the P4B (GATC) fibroblasts to recover de novo synthesis after arresting mtRNA translation using chloramphenicol treatment and release. No changes were observed on the mitochondrial translation, suggesting an accumulation of precursors during chloramphenicol treatment and their higher availability when released (Fig. 5d). These precursors would include mt-tRNA^Gln^ charged with glutamine, allowing for translation to occur at a normal (or close to normal) rate for a few hours (lanes 1, 2, 3; Fig. 5e). However, during continued translation, the rate of charging mt-tRNA^Gln^ with glutamine cannot keep up with the demand, and translation efficiency decreases (lanes 4, 5, 6; Fig. 5e) compared untreated fibroblasts (lanes 7, 8, 9; Fig. 5e). These data demonstrate that the biochemical phenotype becomes more prominent under conditions that place the charging of mt-tRNA^Gln^ under stress.

### Lentiviral rescue experiment

**Fig. 6:**
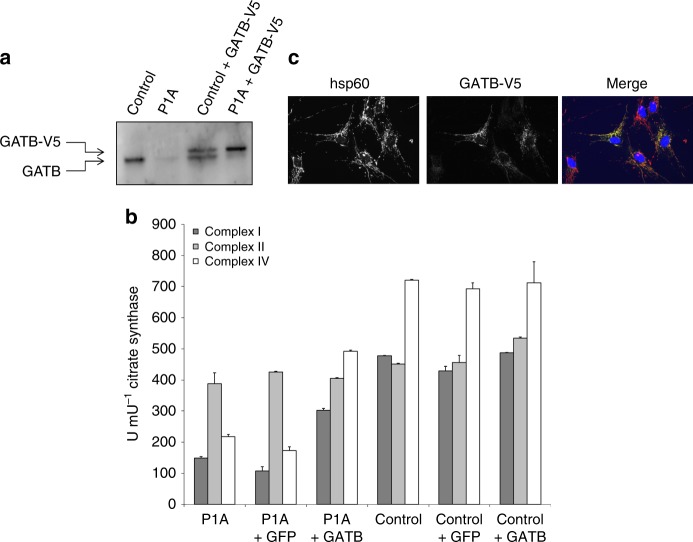
Heterologous expression studies of GatB-V5. **a** Western blot of a SDS-PAGE gel show normal expression of endogenous GatB in the control cell line and near absence of this protein in the patient’s cells, whereas cells lentivirally transduced with GatB-V5 show a band running slightly higher than the endogenous GatB, corresponding to the slightly larger GatB-V5. The expression levels of GatB-V5 in the patient are similar to that of the endogenous GatB in the control cell line. **b** The activities of the respiratory chain enzymes complex I, II, and IV were measured in P1A and control fibroblasts, lentivirally transduced with *GATB*-V5 or GFP (as a negative control), and in non-transduced cells. The results show that the reduced activities of complexes I and IV, presented as mean and standard deviation, as seen in the non-transduced and GFP-transduced patient’s cells, are specifically increased by the transduction of the patient’s cells with *GATB*-V5. There is no effect on complex II. In control cells, the expression of GFP or *GATB*-V5 has no effect on the activity of complex I, II, and IV. From this it is concluded that the enzyme deficiencies in P1A fibroblasts are caused by the pathogenic variants in *GATB*. **c** Subcellular localization study of GatB-V5 in U2OS cells, transiently transfected with a *GATB*-V5 expression construct, and stained using an antibody against V5 (targeting GatB-V5), and hsp60 as a mitochondrial localization marker show variable expression levels of GatB-V5 in different cells, which co-localizes with hsp60 in the merged image on the right-hand side. This shows that the GatB-V5 protein has an exclusively mitochondrial localization

To confirm that the *GATB* gene defect in family 1 was responsible for the respiratory chain enzyme deficiencies in the infant’s fibroblasts, a lentiviral transduction experiment was performed with wild-type *GATB* with a C-terminal V5-tag attached to it. The expression levels of GatB protein obtained after transduction with the *GATB* transgene were very similar to those of the endogenous GatB protein in control cells (Fig. 6a). The expression of the transgene in the affected subject’s fibroblasts resulted in a complete rescue of the respiratory chain enzyme deficiencies, indicating that indeed the *GATB* gene variants in this subject caused the enzyme deficiencies (Fig. 6b). Using the V5-tag, the expression of the transgene could be monitored at the subcellular level. A transient transfection experiment in U2OS cells revealed that the location of the V5-GatB protein was strictly mitochondrial, confirming correct cellular localization and is compatible with its function as part of the mitochondrial GatCAB complex (Fig. 6c).

### Mutation expression in *Saccharomyces cerevisiae*

In addition, to validate the pathogenicity of the *QRSL1* (GATA) and *GATB* mutations identified in the affected subjects we used the yeast *S. cerevisiae* as a model system taking advantage of the presence of the orthologous genes, *HER2* and *PET112*, respectively. GATC was not included in this experiment as yeast lacks an ortholog for this gene.

**Fig. 7:**
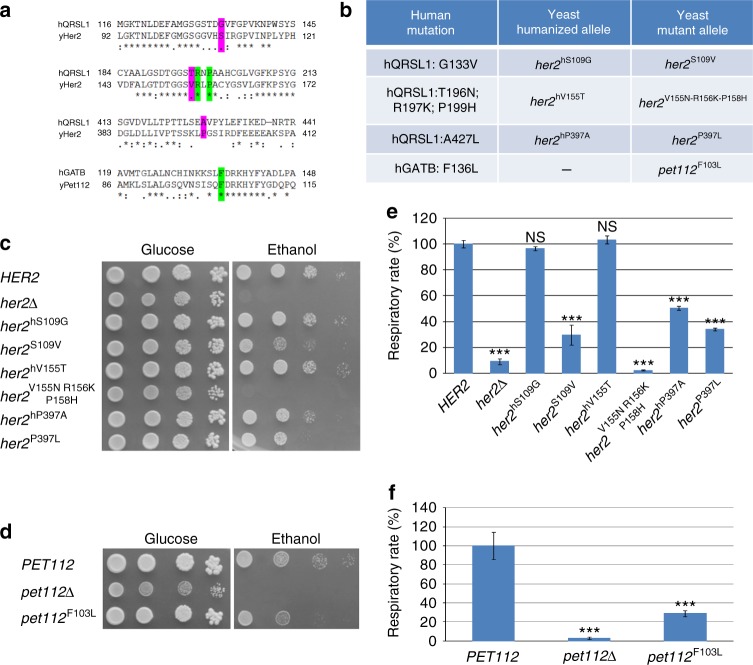
Modeling of the *QRSL1* and *GATB* (*PET112*) variants in *Saccharomyces cerevisiae*. **a** Partial alignment (Clustal Omega) of the human GatA (*QRSL1*) and the yeast Her2 proteins, and the human GatB and yeast Pet112 proteins, respectively. The studied residues are highlighted in green for conserved amino acids, and in magenta for non-conserved. Amino acids are indicated as conserved (*), with strongly similar properties (:) or with weakly similar properties (.). **b** Amino acid changes found in the patients and the corresponding humanized and mutant yeast alleles. **c**–**d** Oxidative growth: the *her2Δ* and *pet112Δ* strains harboring wild-type alleles, humanized or mutant alleles, or the empty vector were serially diluted and spotted on synthetic complete agar plates supplemented with 2% glucose or 2% ethanol in presence (**c**) or in absence (**d**) of the amide donor glutamine, and incubated at 28 °C (**c**) or 37 °C (**d**). High temperature (37 °C) and absence of glutamine were used to exacerbate the *pet112* phenotype. A growth defect in obligatory respiratory medium is evident for the mutants. **e**–**f** Oxygen consumption rates: cells were grown at 28 °C (**e**) or 37 °C (**f**) in SC medium supplemented with 0.6% glucose in presence (**e**) or in absence (**f**) of glutamine. Values were normalized to the appropriate wild-type strain. The data are the results of at least three measurements, and the error bars indicate the standard deviation. Statistical analysis was performed by paired, two-tail Student’s *t* test comparing mutant strains to wild-type (for *PET112*) or humanized (for *HER2*) strains, and the humanized *her*2 strains to the corresponding *HER2* wild-type strain: ***p<0.001, NS not significant

As shown by GATA/Her2 protein alignment (Fig. 7a) the human residues, Arg197 and Pro199, are invariant from human to yeast, corresponding to Arg156 and Pro158 in yeast, respectively. On the contrary, the human amino acid residues Gly133, Thr196, and Ala427 are not conserved in yeast, corresponding in yeast to Ser109, Val155, and Pro397, respectively. For the analysis of pathogenicity of these three non-conserved residues it was necessary to create both the so called humanized version and the potentially pathological allele to compare their effect on mitochondrial function. The humanized version was created by replacing the yeast amino acid with the corresponding amino acid present in the wild-type human allele, as previously described^27^ (Fig. 7b). In order to reveal a possible respiratory growth defect, serial dilutions of the strains were spotted on SC medium supplemented with either glucose or ethanol at 28 °C. The oxidative growth of the strains expressing the humanized versions *her2*^*hS109G*^ and *her2*^*hV155T*^ was similar to that of the wild type (Fig. 7c). The strains expressing the mutant alleles *her2*^*S109V*^ (subject P2A) and *her2*^*V155N-R156K-P158H*^ (subject P3A, paternal allele) show a severely reduced or totally absent oxidative growth. On the contrary, the strain with the humanized allele *her2*^*hP397A*^ shows a slight reduction of growth with respect to the strain with the wild-type version, whereas the strain with mutant version *her2*^*P397L*^ (subject P3A, maternal allele) shows a severe oxidative defective phenotype. Further, the oxygen consumption rate was reduced for all mutants, although to a different extent (Fig. 7e). In particular, parallelizing the growth defect, the mutant *her2*^*V155N-R156K-P158H*^ behaved as the null mutant, whereas the mutants *her2*^*S109V*^ and *her2*^*P397L*^ showed a reduction of the respiratory rate with respect to its humanized version of about 70% and 35% respectively. Overall, the data obtained indicate a pathogenic nature for each of the analyzed variants.

To validate the pathogenicity of the new missense mutation p.Phe136Leu in the *GATB* gene observed in family 1, we introduced the change equivalent to the human mutation into the yeast *PET112* wild-type gene cloned in a centromeric monocopy vector. As shown by protein alignment (Fig. 7a), the human residue Phe136 is invariant in yeast corresponding to Phe103. Oxidative growth and oxygen consumption of the mutant strain *pet112F103L,* measured in the same experimental condition as for the *her2* mutants, did not reveal any defect (Supplementary Fig. 5). We then compared wild-type and mutant strain in a more stressing environmental condition of high temperature (37 °C) and absence of the amide donor glutamine. When grown at 37 °C the mutant displayed a 20% reduction of respiratory activity (Supplementary Fig. 6), whereas at same temperature in the absence of glutamine the mutant showed a defective oxidative growth (Fig. 7d) and a severe (70%) reduction of oxygen consumption (Fig. 7f). These data support the pathogenic role of the Phe136Leu mutation.

In conclusion, pathogenic variants in each of the subunits of the GatCAB complex impair the function of this critical enzyme complex. Decreased GatCAB activity reduces the amounts of glutamine-charged mt-tRNA^Gln^ and disturbs mitochondrial translation for nearly all mtDNA-encoded the OXPHOS subunits. The impact was most evident in highly respiring tissues like the heart, and less so in fibroblasts, a mostly glycolytic tissue with low respiratory needs. Reduced mitochondrial translation and the ensuing effect on respiratory functions correspond to the clinical phenotype, consisting of cardiomyopathy and lactic acidosis with early lethality, regardless of the subunit involved. It remains to be seen if the increased activity in higher cognate amino acid concentrations may guide potential future therapeutic opportunities in disorders of translational defects.

## Methods

### Participants

The study was performed in accordance with the ethical standards of the Declaration of Helsinki. All studies were done in agreement with the rules of the local medical ethics committees. Studies on family 3 were done following IRB approved protocols 07-0386 and 16-0146 approved by the Colorado Multiple Institutional Review Board (COMIRB) at the University of Colorado, and in families 4 and 5 following protocol 0038-14-RMB approved by the Helsinki Committee at Rambam Health Care Campus. Written consent was obtained from all participants that provided samples. Studies on families 1 and 2 were exempt from formal IRB/Ethics board review, as per the local rules at the University of Nijmegen and in Adelaide.

### Exome sequencing

WES was done using previously described methodologies and filtering pipelines as singleton for families 1 and 2^28^, and trio-based for families 3^29^ and 4^30^. In brief, exome enrichment was done using Agilent SureSelect Human All Exon 50 Mb Kit (Agilent Technologies, Santa Clara, CA) for families 1 and 2, Agilent Sure Select All Exon v4 for family 3, and the Nextera Rapid Capture Enrichment kit (Illumina) for family 4, followed by sequencing on a HiSeq2000 sequencer (Illumina, San Diego, CA) for families 1, 2, and 4, and the Illumina HiSeq2500 for family 3. All reads were aligned to the reference genome assembly GRCh37/hg19. Following quality assurance of the reads, the bioinformatics analyses focused on protein-altering variants (missense, nonsense, frameshift, and splice-site). Variants were filtered based on frequency in healthy population databases (dbSNP^31^, 1000Genomes^32^, Exome Aggregation Consortium - ExAc^33^, and NHLBI GO Exome-sequencing Project (evs.gs.washington.edu/EVS), and population-specific databases, such as the Greater Middle-East Variome Project^34^ and the Genome of the Netherlands - GoNL^35^) and in-house databases (<0.5%), and variants were examined using various inheritance models, including dominant (de novo variants), and recessive (compound heterozygous, homozygous, and X-linked hemizygous variants) models. Candidate variants in *QRSL1*, *GATB*, and *GATC* were confirmed by Sanger sequencing. For family 5, Sanger sequencing was performed for presence of the specific *GATC* variant. The following transcripts are used in the description of the candidate variants: *QRSL1* ENSG00000369046 (NM_018292); *GATB* ENST00000263985 (NM_004564), *GATC* ENST00000551765.5 (NM_176818). All missense variants were examined in silico for pathogenicity using the programs PolyPhen 2 (v2.2humvar)^36^, SIFT (JCVI-SIFT v.1.03)^37^, and MutationTaster^38^.

### Respiratory chain enzyme activities and assembly analysis

Fibroblasts were collected from patient P1A at autopsy and from patient P1B from a post mortem skin biopsy. For patient P3A, a full autopsy was performed within one hour after death with heart, liver, and skeletal muscle tissues collected, and a fibroblast culture derived from a skin biopsy. For patients P4A, and P5A, respiratory chain enzyme activities were done on muscle biopsy, and for patient P4B on fibroblasts derived from a skin biopsy. Fibroblasts were routinely cultured in minimum essential medium (MEM)-α with 2 mM glutamine and deoxyribonucleotides (SH3026501 Hyclone) with 10% bovine serum product. For experiments with limited glutamine, fibroblasts were cultured in MEM (M2279 Sigma-Aldrich) with 10% bovine serum product for the stated duration.

The respiratory chain enzyme activities were measured spectrophotometrically in fibroblasts of patients P1A-B and in skeletal muscle, and liver of patient P2A, in Nijmegen as previously described^39^; in muscle, liver, heart, and skin fibroblasts of patient P3A, and fibroblasts of P4B in Colorado, as previously described^40,41^; and in muscle biopsies of patients P4A and P5A in Israel, as previously described^42^. Additionally, isolated mitochondrial membrane fractions of muscle, heart, liver, and fibroblast samples of P3B, and fibroblasts of P4B were analyzed by BN-PAGE with in-gel activity staining^40,41,43^, and the assembly of complex I in the P3B patient was assessed by western blotting following non-denaturing BN-PAGE of isolated mitochondrial membrane fractions of muscle, heart and fibroblasts using an antibody for NDUFS2, a component of the earliest complex I assembly intermediate^29^.

### Protein analysis by western blot

Western blot analysis was performed on mitochondria isolated by differential centrifugation from the patients' skeletal muscle, heart muscle, liver, and cultured skin fibroblasts, and detected using the appropriate HRP-conjugated secondary antibody followed by enhanced chemiluminescence in families 1–3, using antibodies for GatA, citrate synthase, GatB, GatC, green fluorescent protein, and for V5. The sources of all antibodies used in this study are listed in Supplementary Table 3.

Fibroblast pellets were incubated in RIPA buffer containing protease inhibitors followed by centrifugation, separation of proteins on SDS-PAGE gels, immunoblotted, and detected with the primary antibodies with quantitative analysis using ImageJ software using antibodies against MT-COXI, GatA, GatB, GatC, VDAC1, total human respiratory chain western blot antibody cocktail, which contains antibodies against complex I subunit NDUFB8, complex II subunit 30kDa, complex III subunit core 2 (UQCRC2), complex IV subunit II (COX II), and ATP synthase subunit-α (ATP5A1). The assembly of the glutamyl-tRNA (Gln) amidotransferase complex was analyzed on a native gel followed by western blotting using an anti-GatB antibody. This analysis also provided an estimation of the size of the complex, and a possible super complex. All the uncropped western blots with molecular weight indicated are included in Supplementary Figures 7–16.

### mRNA analysis

Levels of mRNAs of the different GatCAB subunits were analyzed by RT-qPCR. These assays were carried out on RNA extracted from fibroblasts using HPRT levels for normalization, and two primer pairs were used for each PCR target (primers are available upon request).

### Respirometry assays

Cellular oxygen consumption was measured by high-resolution respirometry on an Oroboros Oxygraph2k, using a SUIT protocol^29^ for patients P3A and P4B, with experiments done in fibroblasts grown in glutamine-containing MEM-α and in minimal MEM tissue culture media.

### Aminoacylation assay

The impact of candidate variants in the GatCAB complex on the aminoacylation of mt-tRNA^Gln^ was analyzed using RNA isolated from fibroblasts grown in standard tissue culture medium supplemented with Glutamax, and/or in fibroblasts grown in MEM without glutamine^44^. RNA was extracted from subconfluent fibroblasts using Trizol (Life Technologies), and the final RNA pellet was dissolved in 10 mM sodium acetate, pH 5.0 at 4 °C. For the deacylated (dAc) control, the pellet was resuspended in 200 mM Tris-HCl at pH 9.5 and incubated at 75 °C for 5 min, followed by RNA precipitation and resuspension in 10 mM sodium acetate buffer pH 5. Next, 5 µg of RNA was separated on a 6.5% polyacrylamide gel (19:1 acrylamide:bisacrylamide) containing 8 M urea in 0.1 M sodium acetate pH 5.0 at 4 °C and blotted to Hybond N+ membranes (Amersham RPN303B). Following UV-crosslinking, the blots were washed in hybridization buffer of 7% SDS, 0.25 M sodium phosphate pH 7.6 for 1 h at 65 °C. The membrane was subsequently incubated overnight at 65 °C in hybridization solution with ^32^P-labeled antisense RNA probes, generated by in vitro transcription by T7 RNA polymerase in the presence of ^32^P-labeled alpha-UTP, using linearized templates. After hybridization, the blots were washed six times with 1x SSC for 15 min at 65 °C. Bound probes were detected by phosphor imaging on an Amersham Typhoon Scanner.

### Pulse labeling of mitochondrial translation products

In vivo labeling of mitochondrial translation products was carried out as previously described^6^. Exponentially growing 2.5x10^5^ fibroblasts were labeled for 90 min in methionine and cysteine-free DMEM containing 200 μCi ml^−1^ of [S^35^]-methionine (Perkin Elmer) and 100 μg ml^−1^ of the cytoplasmic protein synthesis inhibitor emetine. Cells were harvested and total cellular protein was extracted from which 50 μg were loaded on 17.5% protein denaturing gels. After electrophoresis, gels were fixed, incubated in Amplify Fluorographic Reagent (GE Healthcare, ref. NAMP100) for 20 min and dried. The labeled mitochondrial translation products were detected by direct autoradiography.

### Depletion and recovery of mitochondrial proteins

In order to analyze the recovery kinetics of respiratory chain proteins after depletion, mtDNA-encoded peptides were eliminated by treatment with 50 μg.ml^−1^ of the mitochondrial translation inhibitor chloramphenicol for six days. The depletion of mtDNA-encoded respiratory chain subunits impairs the assembly of respiratory chain complexes I, III, IV, and V, which results in a depletion of the nuclear encoded respiratory chain proteins identified in the human western blot antibody cocktail described above. To analyze the recovery of complex I, III, and IV subunits, chloramphenicol containing media was removed, cells were washed twice with PBS, and incubated with chloramphenicol-free media. Cells were then collected at 0, 10, 16, 24, and 48 h after chloramphenicol removing, and analyzed by western blot. In this experiment, the recovery of complex I NDUFB8, Complex III subunit Core 2 and complex IV COX II is seen. Complex II, which is nuclear encoded, acts as a control. Since the complex V subunit alpha belongs to F1-ATP synthase, which assembles in the absence of mitochondrial subunits, it is not depleted by chloramphenicol treatment^45^. To examine the recovery of mitochondrial protein synthesis after chloramphenicol treatment, stressed by absence of cytoplasmic protein translation, cells were washed and transferred to [S^35^]-methionine and emetine-containing medium, and processed for autoradiography above.

### Transfection experiments

A full-length cDNA (NM_004564.2) with or without a stop codon was cloned into a pDONR201 vector, and recombined with pLenti6.2⁄V5-DEST Gateway Vector, using the Gateway LR Clonase II enzyme mix (Invitrogen), and resulting in a lentiviral expression construct for V5-tagged GatB. HEK293FT cells were transfected to produce lentivirus according to the manufacturer’s protocol (Invitrogen). The culture medium containing lentiviral particles was harvested after 72 h, and was added to the patient and healthy control fibroblasts. The next day, the virus was removed, and the medium was refreshed. After 48 h, 2.5 μg.ml^−1^ blasticidin was added to the medium to select for the transfected cells, and after 14 days culture on selection medium, the blasticidin-resistant cells were used for analysis. As a control, fibroblasts were transduced with a GFP-V5 lentivirus. Expression of the transgenes was checked by western blot analysis using anti-GFP and anti-V5 antibodies, and respiratory chain and citrate synthase enzyme activities were measured. To examine the subcellular localization, the pLenti6.2 plasmid with GatB-V5 was transfected to U2OS cells, and after 48 h incubation, cells were stained using an anti-V5 and anti-HSP60 antibody, and detected by confocal microscopy.

### QARS cellular localization experiments

A human cDNA for *QARS* was obtained from Source Bioscience (IMAGE ID 2821788), and following sequence verification, the cDNA was amplified by PCR and cloned into the pmaxGFP plasmid (Lonza) in-frame with the GFP open reading frame using Gibson Assembly Master Mix (New England Biolabs). In order to generate a positive control for mitochondrially localized GFP, a mitochondrial targeting sequence coding for the first 49 amino acids from subunit F1β of human mitochondrial ATP synthase was also cloned into pmaxGFP. The resulting plasmid DNA was transiently transfected into HeLa cells grown to 50–60% confluence in DMEM, containing 4.5 g l^−1^ glucose, 110 mg l^−1^ sodium pyruvate, and supplemented with 10% FBS, 100 U.ml^−1^ penicillin and 100 μg.ml^−1^ streptomycin using Lipofectamine 2000 (Thermo Fisher Scientific), according the manufacturer’s instructions. Transfected cells were fixed, permeabilized, and immunostained with anti-TOM20 primary antibody and Goat Anti Rabbit Alexa Fluor 594 secondary antibody^46^. To delineate the cell border, the cells were additionally stained with CellTracker Blue CMAC Dye (Thermo Fisher Scientific), according to manufacturer’s instructions and with DAPI to stain the nucleus. Images were acquired using an A1R-Si Nikon N-SIM confocal microscope. In order to further localize the endogenous QARS protein, cell fractionation was performed^47^, and the QARS protein was detected by western blotting with NDUFB8 and GAPDH as controls.

### Yeast strains, plasmids, and media

The W303-1B genotype (obtained from ATCC, catalog # 201238) is *Matα ade2-1 leu2-3, 112 ura3-1 trp1-1 his3-11, 15 can1-100*. All experiments except transformation were performed in synthetic complete (SC) media (0.69% YNB without amino acids powder, ForMedium) supplemented with 1 gr l^−1^ dropout mix^48^ without amino acids or bases necessary to keep plasmids (i.e., uracil for pFL38 and tryptophan for the pFL39). Media were supplemented with various carbon sources at 2% (w/v) (Carlo Erba Reagents), in liquid phase or after solidification with 20 g l^−1^ agar (ForMedium). Transformation was performed after growth in YPAD medium (1% Yeast extract, 2% Peptone, 40 mg l^−1^ adenine base and 2% glucose)^49^. *HER2* and *PET112* were cloned under their natural promoters by PCR-amplification and inserted into the pFL38 vector^50^. The pFL38-*HER2* or pFL38-*PET112* plasmid was introduced into the W303-1B strain through the Li-Ac method^51^ and disruption of the genomic *HER2* or *PET112* gene was performed in this strain, since the deletion leads to mitochondrial DNA loss^51,52^. The disruption was performed through one-step gene disruption by PCR-amplification of KanMX4 cassette^53^ from the BY4742 deleted strain using appropriate primers and transformation of the former strain; thus obtaining W303-1Bh*er2Δ*/pFL38-*HER2* and W303-1B*pet112Δ*/pFL38-*PET112*. *HER2* and *PET112* fragments were subcloned from pFL38 to pFL39^50^. *HER2* and *PET112* were mutagenized by PCR overlap technique^54^ with appropriate primers to obtain the humanized and mutant alleles, and subsequently they were cloned into the pFL39 vector. W303-1B*her2Δ*/pFL38-*HER2* was transformed with the pFL39 vector carrying the wild-type (*HER2*), or the humanized (*her2*^*hS109G*^ or ^*her2hV155T*^ or *her2*^*hP397A*^), or the mutant (*her2*^*S109V*^ or *her2*^*V155N-R156K-P158H*^ or *her2*^*P397L*^) alleles, or with the empty vector as control, and then pFL38-*HER2* was lost through plasmid-shuffling. W303-1B*pet112Δ*/pFL38-*PET112* was transformed with pFL39-*PET112*, pFL39-*pet112*^*F103L*^ or the empty vector pFL39, and then pFL38-*PET112* was lost through plasmid-shuffling. To evaluate mitochondrial respiratory activity in yeast, the oxygen consumption rate was measured at 30 °C from yeast cell suspensions cultured for 18 h at 28 °C or for 16 h at 37 °C in SC medium supplemented with 0.6% glucose until exhaustion using a Clark-type oxygen electrode (Oxygraph System Hansatech Instruments England) with 1 ml of air-saturated respiration buffer (0.1 M phthalate–KOH, pH 5.0), 0.5% glucose.

### Structural modeling of the human GatCAB complex

Amino acid sequences of the GatC (O43716), GatA (Q9H0R6), and GatB (O75879) subunits were retrieved from UniProt^55^ and parsed through SWISS-MODEL^56^ to search for modeling templates. Templates were chosen considering coverage and identity: GatC was modelled to the structure of the corresponding subunit in the *Staphylococcus aureus* complex (PDB 2G5H)^16^; GatA was modelled using the subunit of *Aquifex aeolicus* (PDB 3H0M)^57^; GatB was modelled using the high-resolution structure from *Staphylococcus aureus* (PDB 3IP4)^58^. Finally, all models were aligned to the bacterial GatCAB complex from *Thermotoga maritima* in the glutamylation state (PDB 3AL0)^59^ using PyMOL^60^. The human GatCAB model was manually inspected for clashes. The magnesium ion coordinated to the amidotransferase centre was present in the structure of GatB from *Thermotoga maritima*, while ADP and the magnesium ion coordinated by its phosphate groups were obtained by aligning the structure of GatB from *Aquifex aeolicus* (PDB 3H0R)^57^ to the corresponding modelled subunit. Residues relevant for the catalytic activity at the amidotransferase site were mapped from the *Saccharomyces cerevisiae* GatFAB complex.^61^ Structure visualisation and mapping of residues were performed using PyMOL^60^.

### Statistics

As needed, comparison between two groups was done by two sided Student *t*-test paired or unpaired as indicated,with calculations done in either Excel (Microsoft) or SPSS 24 (IBM).

## Electronic supplementary material

Supplementary Information

## Data Availability

The molecular data are deposited in the ClinVar database (www.ncbi.nlm.nih.gov/clinvar/), with accession numbers listed in Supplementary Table 4. All other data supporting the findings of this study are available within the paper and the Supplementary information. Quantitative data associated with Figs. 4, 6, and 7 are available upon request.
