# Supplementary material for: Pathogenic variants in glutamyl-tRNAGln amidotransferase subunits cause a lethal mitochondrial cardiomyopathy disorder
Source: Nat Commun. 2018 Oct 3;9:4065. doi: 10.1038/s41467-018-06250-w (PMC6170436; doi:10.1038/s41467-018-06250-w)
Supplement: Supplementary file 1 — Supplementary Information [file 41467_2018_6250_MOESM1_ESM.pdf]

Pathogenic variants in glutamyl-tRNA^{Gln} amidotransferase subunits cause a lethal mitochondrial cardiomyopathy disorder

Supplementary Note 1

Clinical descriptions

Family 1

Patient 1A (II-1): The male proband was born as the first child of non-consanguineous healthy parents of Caucasian descent (Figure 1b). At a gestational age of 34 weeks he had fetal bradycardia (90.min^{-1}, low variability) in combination with fetal hydrops, characterized by ascites, cardiomegaly, and pericardial effusion with a normal structural heart on prenatal ultrasound. Prenatal exam showed a normal karyotype and no signs of fetal anemia. Because of fetal distress and increasing hydrops, a Caesarean section was performed at 35 weeks gestational age. Apgar score was 2 after 1 minute, and the boy was intubated immediately because of respiratory failure. There was an asymmetric growth restriction with birth weight of 2,030 g (-2.2 SD), and a head circumference of 31 cm (-1.4 SD). Due to the hydrops, dysmorphic features were difficult to assess, but two preauricular tags and neonatal teeth were present. The boy had anemia with an initial hemoglobin concentration of 10.5 g.dL^{-1}. Investigations for TORCHES and Parvo-B virus were negative but the Kleihauer-Betke test was positive (0.59 mL; normal: 0-0.01 mL), indicating a feto-maternal transfusion. The amplitude integrated EEG displayed burst suppression, possibly due to perinatal asphyxia. After initial stabilization, the clinical condition deteriorated rapidly. There was severe cardiac dysfunction on echocardiogram with impaired cardiac contractility and pericardial effusion, treated with

inotropes, and persistent pulmonary hypertension treated with nitric oxide. Echocardiogram showed bradycardia, thickened myocardium primarily in the right atrium and atrial septum and moderately decreased contractility of both ventricles, and signs of pulmonary hypertension. Adrenal function was normal with cortisol 245 nmol.L^{-1} (normal 150-500 nmol.L^{-1}). Prominent metabolic acidosis with lactate 22.2 mmol.L^{-1} evolved. Despite intensive care treatment, the boy died on the second day of life. Autopsy revealed massively increased myelopoiesis and erythropoiesis, pulmonary hypoplasia and cardiomegaly (Figure 1h). Lysosomal storage disease testing in fibroblasts was negative.

Patient 1B (II-3): After a healthy daughter had been born for these same parents, this third pregnancy was complicated by intrauterine growth retardation, cardiomegaly and pericardial effusion. From 30 weeks gestational age on there was relative bradycardia of 110.min^{-1}, and from 33 weeks gestational age a pericardial effusion was present without signs of cardiac decompensation or other signs of hydrops. Investigations for TORCHES serology and SS-A and SS-B antibodies were negative. After a Caesarean section at a gestational age of 37 weeks, a girl was born with Apgar scores 3, 3, 7 after 1, 5, and 10 minutes, respectively. Respiratory insufficiency rapidly necessitated artificial ventilation. There was an asymmetric growth restriction with a birth weight of 1,525 g (-5.6 SD), length of 43 cm (-4 SD), and a head circumference of 29 cm (-3.8 SD). The infant had no dysmorphic features, and only mild peripheral edema. Heart rate was low at 105.min^{-1} and cardiac ultrasound revealed biventricular impaired cardiac contractility with a shortening fraction of 27% but no dilation (left ventricular diameters at the 90th percentile), and mild pericardial effusion. There was anemia with a hemoglobin concentration of 10.6 g.dL^{-1}. Initially there was hypoglycemia 19.8

mg.dL^{-1} at 2 hours after birth, which normalized within 4 hours after birth with 7.6 mg.kg^{-1}.min^{-1} intravenous glucose administration. Blood lactate levels increased from 21.6 mmol.L^{-1} to 33.6 mmol.L^{-1}. In subsequent hours vital functions could not be stabilized despite intensive ventilatory and inotropic support, and carnitine supplementation, and she died within 24 hours after birth. Metabolic investigations of plasma acylcarnitines and sialotransferrin profile were normal, and urine organic acid analysis showed only massive lactate excretion. A microarray was normal. A respiratory chain defect was clinically suspected. Clinical sequencing did not identify pathogenic variants in mtDNA, or in the *TUFM*, *TSFM*, *GFM1*, *GFM2*, *SCO2*, or *POLG* gene. An autopsy was declined.

Family 2

Patient 2A (II-2): The proband was the second child to non-consanguineous parents of Caucasian and Japanese descent (Figure 1c). Their previous child was a healthy male. The proband was born by normal delivery after an uneventful pregnancy, and only had a brief admission for transient hypothermia with normal glycemia. He had normal newborn screening but failed his hearing screen and had bilateral profound sensorineural hearing loss. At 3 months of age, he presented acutely unwell with fever followed by encephalopathy, hypotonia, and poor perfusion. He was hypoglycemic with metabolic lactic acidosis pH 7.23, bicarbonate 6.3 mM, anion gap 25.3 mmol.L^{-1}, BE -20 mEq.L^{-1}, and lactate 14.9 mM. There was hepatomegaly confirmed on ultrasound, with mildly elevated transaminases AST 89 IU.L^{-1}, γ-GT 304, and abnormal clotting with an INR 1.4 (normal 0.9-1.3), aPTT 57 sec (normal 28-41), fibrinogen 0.88 g.L^{-1} (1.80-4.0) without D-dimers. Hemoglobin was 10 g.dL^{-1} (normal 10-14). After normalization of the glycemia, fluid status and cardiovascular status, the lactic acidosis persisted, albeit

improved (lactate 6.4 mM), and he remained lethargic. Multiple viral studies, ammonia, lipid, and α1-antitrypsin were normal. Urine organic acids showed ketonuria, lactate and pyruvate, Krebs cycle intermediates, and p-hydroxyphenyllactate and p-hydroxyphenylpyruvate. Amino acids in plasma showed elevated alanine and glutamine. His general condition improved clinically sufficient to begin feeding. After 48 hours, he suffered a sudden cardiac arrest and could not be resuscitated. Autopsy showed cardiomegaly, pericardial and pleural effusions, and hepatic steatosis. On electron microscopy, there were megamitochondria with tightly packed cristae.

Family 3

Patient 3A (II-3): The proband was the product of the third pregnancy to non-consanguineous Caucasian parents (Figure 1d). A previous pregnancy resulted in intrauterine demise at 25 weeks with a severely hydropic fetus of unknown etiology but with a normally sized heart. Maternal hypothyroidism was adequately treated throughout pregnancy. The pregnancy of the proband was complicated at 19 weeks with the fetus showing a dilated heart with ventricular wall thickening, decreased biventricular function, and pericardial effusion. Infectious causes were excluded including using pericardiocentesis; karyotype and microarray were normal, as was Pompe disease enzyme activity. The mother was treated with intravenous immunoglobulin. Severe fetal anemia was identified and treated with fetal red blood cell transfusions given at 28, 29, and 30 weeks gestation. There were no hemoglobin abnormalities and no signs of immune-mediated hemolysis. Due to worsening fetal biventricular cardiac dysfunction, ascites and pericardial effusion, this girl was born at 31 weeks gestation by urgent Caesarean section. She was intubated and ventilated shortly after birth for poor respiratory

effort. Birth weight was 1.68 kg (+0.59 SD), length 39 cm (+1.44 SD), and head circumference 30 cm (-0.33 SD), all within normal range. At birth, she had a hematocrit of 28% hemoglobin 8.9 $g.dL^{-1}$ (normal 11.6-16.6), platelets $84,000.mm3^{-1}$ (normal 150-500), and she received a red blood cell transfusion. She developed rapidly worsening lactic acidosis from lactate 14 mM at 2 h of life progressing to 24 mM, and with a lactate/pyruvate ratio elevated at 77 increasing to 99, and with pH remaining below 7.0 despite treatment. She had ascites which was drained, and increased PT (INR 3.1), prolonged PTT 129 sec (normal 29-48 sec), and D-dimers present, elevated troponin and increased creatine kinase (CK) levels 413. On echocardiogram, she had a noncompacted myocardium, biventricular hypertrophy, and pericardial effusion, and on X-ray cardiomegaly. Initially ventricular function was preserved with high inotropic support for low blood pressures. Cortisol was 1.2 $\mu g.dL^{-1}$ (normal 1.7-14.1), and hydrocortisone was given. Biventricular function declined rapidly with worsening acidosis, and the infant died at the end of the first day of life.

Testing for multiple viruses and infectious causes was negative. She had normal carnitine levels and a normal acylcarnitine profile. Mitochondrial DNA sequencing and quantification of mitochondrial DNA in muscle (77% of mean control value) were normal. A clinical nextgen sequencing of a panel of 666 nuclear genes related to mitochondrial disease identified no pathogenic variants, other than a single heterozygous variant in *MARS2* (c.1580T>C p.Val527Ala) affecting a conserved amino acid and in *RARS2* (c.1123G>A p.Val375Met), with no second variant found and no exonic copy number change such as a deletion identified. A rapid autopsy was done with samples obtained within one hour after her death. The autopsy showed a very marked cardiomegaly (heart weight 39.2 g, normal value for

gestational age 9 ± 2.8 g) with biventricular thickening of both atria (right more than left) and ventricles. On microscopic examination, there was no cardiomyocyte hypertrophy but cardiomyocyte perinuclear clearing (Figure 1g). On electron microscopy, there was extensive mitochondrial proliferation with swelling and poor inner mitochondrial membrane, and decreased contractile elements (Figure 1j). Skeletal muscle showed rare hypertrophied fibers and more pronounced mitochondrial staining, and electron microscopy showed ballooned rarefied mitochondria without inclusions. The lungs were small 23.8 g (normal 28.5 g) with hyaline membranes and modest intima thickening of arterioles. There were no sideroblasts either in the bone marrow or in the extramedullary hematopoiesis present in the liver.

Family 4

Three infants, two males and a female, were born to parents who were first cousins of Druze origin following full term pregnancies (Figure 1e). Pregnancies were described as uneventful and initial development was normal. All three children presented at ages 2-3 months with cyanosis and respiratory deterioration.

Patient 4A (II-1) – The proband had reportedly normal development until age 3 months, when he was hospitalized due to failure to thrive. Several hours after hospitalization he developed cyanosis, hyperventilation and hypotension. He was transferred to the pediatric intensive care unit (PICU), but died following several recurrent similar severe events. Laboratory findings included severe lactic acidosis pH 7.26, lactate 4 to 12 $mmol.L^{-1}$ (normal < 1.8), anemia with hemoglobin 8.1 $g.dL^{-1}$ (normal: 11.1-14.1), elevated creatine phosphokinase (CPK) 793 $U.L^{-1}$ (normal <250), and transaminase AST 129 $U.L^{-1}$ (normal 5-40). Blood amino acids showed high alanine 649 $\mu mol.L^{-1}$ (normal 170-600), and urine for organic acids revealed only high lactate

and ketones. Echocardiography showed combined dilated and hypertrophic cardiomyopathy. A cardiac muscle specimen, taken post mortem, showed vacuolation of the cardiomyocytes (Figure 1i), staining strongly with SDHB indicating mitochondrial proliferation (Figure 1l), which was also evident on electron microscopy with displacement of contractile elements (Figure 1k). Skeletal muscle biopsy was taken for mitochondrial studies.

Patient 4B (II-3) – The boy had reportedly been healthy, with no symptoms of cardiac disease or failure to thrive, until at age 2 months, when during routine echocardiography he developed cyanosis and respiratory distress necessitating mechanical ventilation. Echocardiography demonstrated concentric hypertrophy with an echo-bright endocardium and reduced global function. Laboratory findings showed severe acidosis, with intermittently high serum lactate levels up to 14 mmol.L^{-1}, although cerebrospinal fluid lactate was normal. He also had elevated creatine phosphokinase 938 U.L^{-1}, and elevated liver enzymes AST, ALT and LDH. His alanine was elevated 850 µmol.L^{-1} with branched amino acids slightly above the normal range, and urine organic acids showed high lactate and ketones. No defects in β-oxidation of fatty acids were observed in lymphocytes. The child was stabilized and treated with captopril, digoxin, carnitine and thiamine. Physical examination revealed a systolic 2/6 murmur at the left sternal border, he had normal muscle tone and no hepatomegaly or splenomegaly. Following repeat episodes of cyanosis and respiratory failure, the child died. Pyruvate dehydrogenase activity was normal.

Patient 4C (II-5) – The girl was born following full-term pregnancy; prenatal echocardiography was normal. She was reportedly healthy until her first cyanosis event during feeding at age 3 months. Chest X-ray was normal. At age 3.5 months, a recurrent cyanotic event left the child

unconscious with severe lactic acidosis with lactate 6.1 to 15.7 $mmol.L^{-1}$, creatine phosphokinase 998 to 1877 $U.L^{-1}$, troponin 4.29 $ng.mL^{-1}$ (normal <0.28), and elevated liver enzymes LDH, AST, and GGT. Echocardiography revealed concentric hypertrophy of the left ventricle with reduced left ventricular function. The child died after several days of intensive care treatment.

Family 5

The parents are healthy first-degree cousins of Druze origin (Figure 1f), with no known relation to Family 4, but residing in an adjacent village. The family history revealed a paternal aunt (I-2) who had died at the age of 4 months from progressive cardiomyopathy and pleural effusion. She, reportedly, had reduced respiratory complexes I and IV on muscle biopsy.

Patient 5A (II-2) - The proband was born at week 33 as part of a twin gestation, with his twin succumbing within one hour after birth from a congenital abdominal wall defect. He was born with a low Apgar score, but quickly recovered and had normal development thereafter. At age 5.5 months he exhibited reduced eating and general deterioration; several days later he was hospitalized due to cardiomyopathy and pericardial effusion, and required mechanical ventilation. Echocardiography revealed severe concentric hypertrophic cardiomyopathy. Laboratory workup revealed metabolic acidosis with pH 7.27, elevated blood lactate 8 $mmol.L^{-1}$ which later normalized, elevated creatine phosphokinase 660-1146 $U.L^{-1}$, elevated troponin and anemia with hemoglobin 9.2 $g.dL^{-1}$ (normal 11-14). He had mildly elevated alanine 649 $\mu mol.L^{-1}$ (normal 170-600), and on urine organic acid high lactate, succinate, fumarate, 3-methylglutaconate and ketones. A mitochondrial disorder was suspected. He was treated with pericardiocentesis, and inotropes, which resulted in mild improvement. He was discharged on

captopril and diuretics. He returned to the hospital after one month with cough, oxygen desaturation and fever. Repeat echocardiography revealed combined dilated and hypertrophic cardiomyopathy. During this hospitalization, his respiratory and metabolic state deteriorated and he eventually died at age 6.5 months. Skeletal muscle biopsy was obtained postmortem for mitochondrial studies.

Patient 5B (II-3) - In a subsequent pregnancy, the parents chose not to pursue prenatal diagnosis, and were followed up with fetal echocardiograms, which were normal. A girl was born by Caesarean section. Development and echocardiography testing remained normal until age 3 months, when asymptomatic mild concentric cardiomyopathy, more prominent in the left ventricle, was first detected; no signs of heart failure were observed. At age 5 months she was asymptomatic, with normal development and weight gain, but echocardiography revealed considerable left ventricle and septum hypertrophy with normal systolic function and mild pericardial effusion. Treatment with furosemide was started. She had elevated lactate 18 $mmol.L^{-1}$; but normal creatine phosphokinase and transaminases. During a genetics evaluation at 6 months, she was tachypneic with perioral cyanosis, but no other signs of heart failure. Within 10 days, she stopped feeding and showed breathing difficulties requiring hospitalization; she died suddenly the next day despite attempted resuscitation.

Supplementary Figures

Supplementary Figure 1: Conservation of amino acids observed in patient with pathogenic variants in genes that are components of the GatCAB complex.

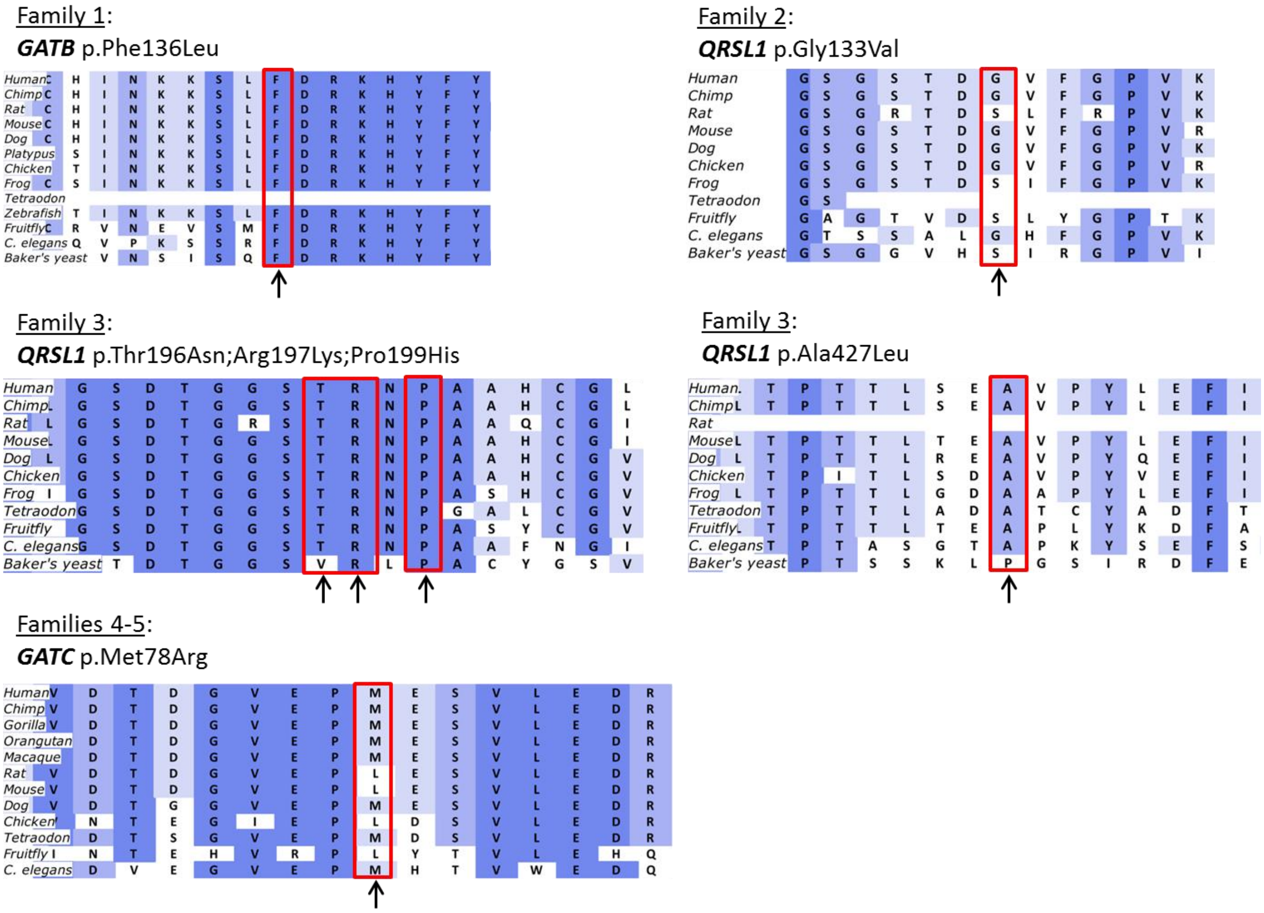

Legend: Conservation of the missense mutated amino acids in the genes *QRSL1*, *GATB*, and *GATC* in the species *Homo sapiens* (human), *Pan troglodytes* (chimpanzee), *Rattus norvegicus* (rat), *Mus musculus* (mouse), *Canis familiaris* (dog), *Gallus gallus domesticus* (chicken), *Xenopus tropicalis* (frog), *Tetraodon nigroviridis* (pufferfish), *Drosophila melanogaster* (fruitfly), *Caenorhabditis elegans* (worm) and *Saccharomyces cerevisiae* (Baker's yeast) is derived with Alamut 2.5 software.

Supplementary Figure 2: mRNA levels of subunits of the GatCAB complex.

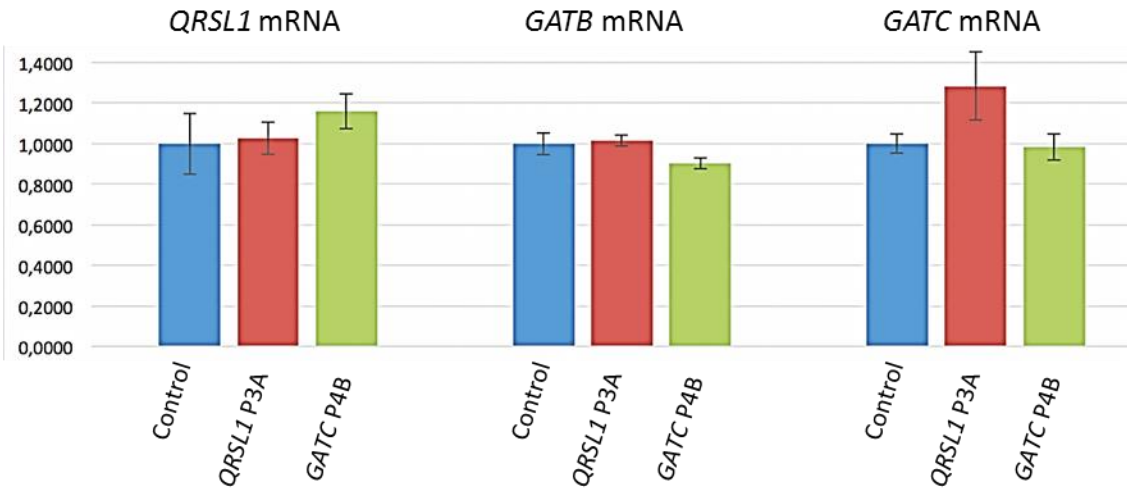

Legend: The amount of the mRNA is determined by qPCR in exponentially growing fibroblasts. RNA was extracted with Trizol reagent (Sigma), and cDNA synthesized using the QuantiTect Reverse Transcription Kit (Qiagen), followed by SYBR green RTqPCR using an Applied Biosystems Step-One Plus real-time thermocycler (Applied Biosystems, ThermoFisher), consisting of an initial 10 min denaturation step at 95°C followed by 40 cycles of denaturation (15 sec at 95°C) and annealing/extension (1min at 60°C). The mRNA relative copy numbers were determined by qPCR normalizing to HPRT levels, using for each PCR target two primer pairs (see Supplementary methods). Mean and standard deviation are shown.

Supplementary Figure 3: Cellular localization of QARS.

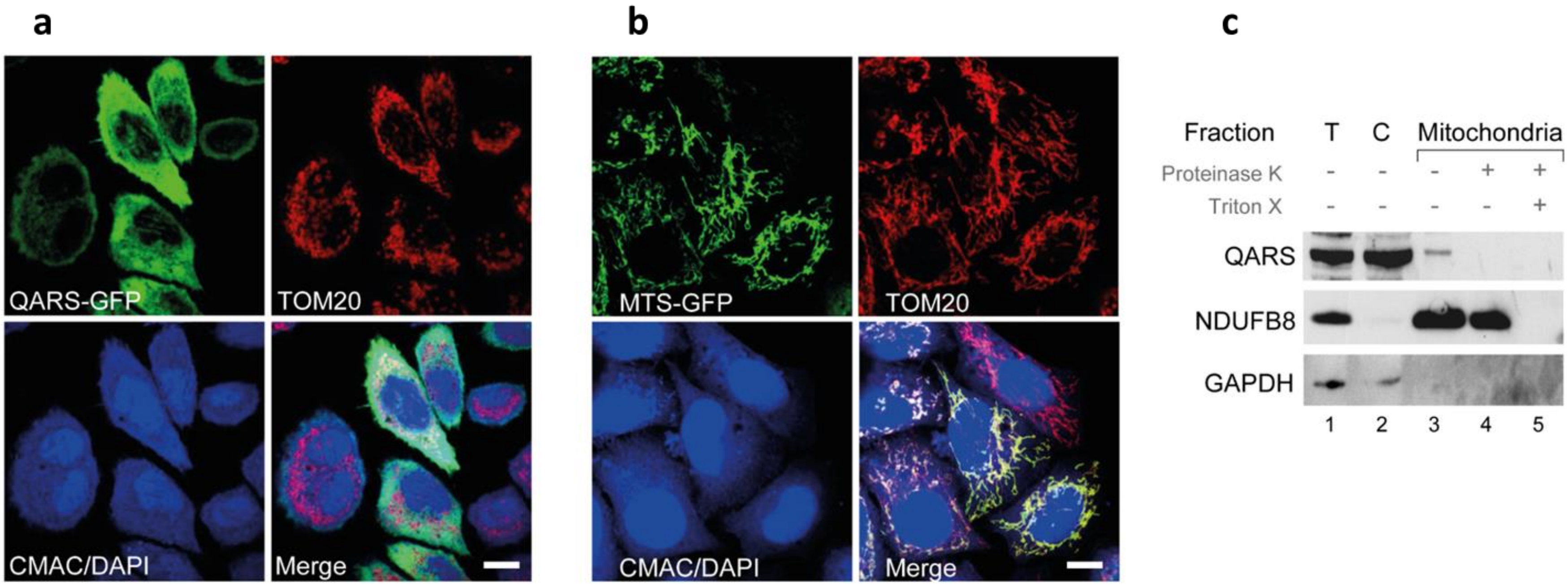

Legend: (a) Cellular localization of the QARS-GFP construct (green) analyzed by confocal microscopy. Cells were counterstained for the mitochondrial import protein TOM20 (red). CMAC and DAPI were used to stain the cytoplasm and cell nuclei, respectively. Scale bar - 10 micrometers. (b) Cellular localization of the mitochondrially targeted GFP (MTS-GFP, green), used here as a control, analyzed by confocal microscopy. Cells were counterstained for the mitochondrial import protein TOM20 (red). CMAC and DAPI were used to stain the cytoplasm and cell nuclei, respectively. Scale bar - 10 micrometers. (c) HeLa cells were fractionated into cytosol ("C", lane 2) and mitochondria ("M", lanes 3-5). The mitochondrial fraction was treated with proteinase K (lane 4) or proteinase K and Triton X (lane 5). "T" (lane 1) depicts total cell extraction. Sub-cellular fractions were analyzed by western blotting with antibodies against QARS, NDUFB8 (mitochondrial complex I) and GAPDH (cytosol).

Supplementary Figure 4: mRNA levels of nuclear encoded OXPHOS subunits.

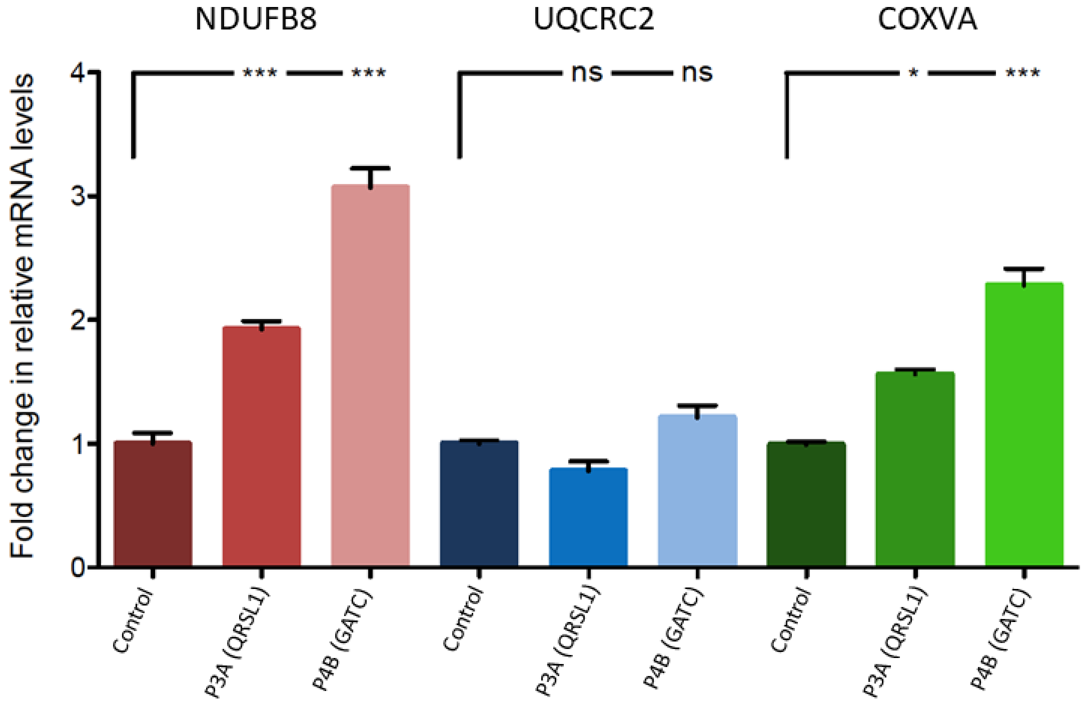

Legend: RT-qPCR analysis of NDUFB8, UCQCR2 and COX5A mRNA levels in indicated samples, normalized to HPRT levels. Shown are the fold changes in relative mRNA levels as mean ± standard deviation for triplicate samples, compared to the control set to 1.0. *, p value < 0.05; ***, p value < 0.001; ns = not significant.

Supplementary Figure 5: pet112 phenotypes.

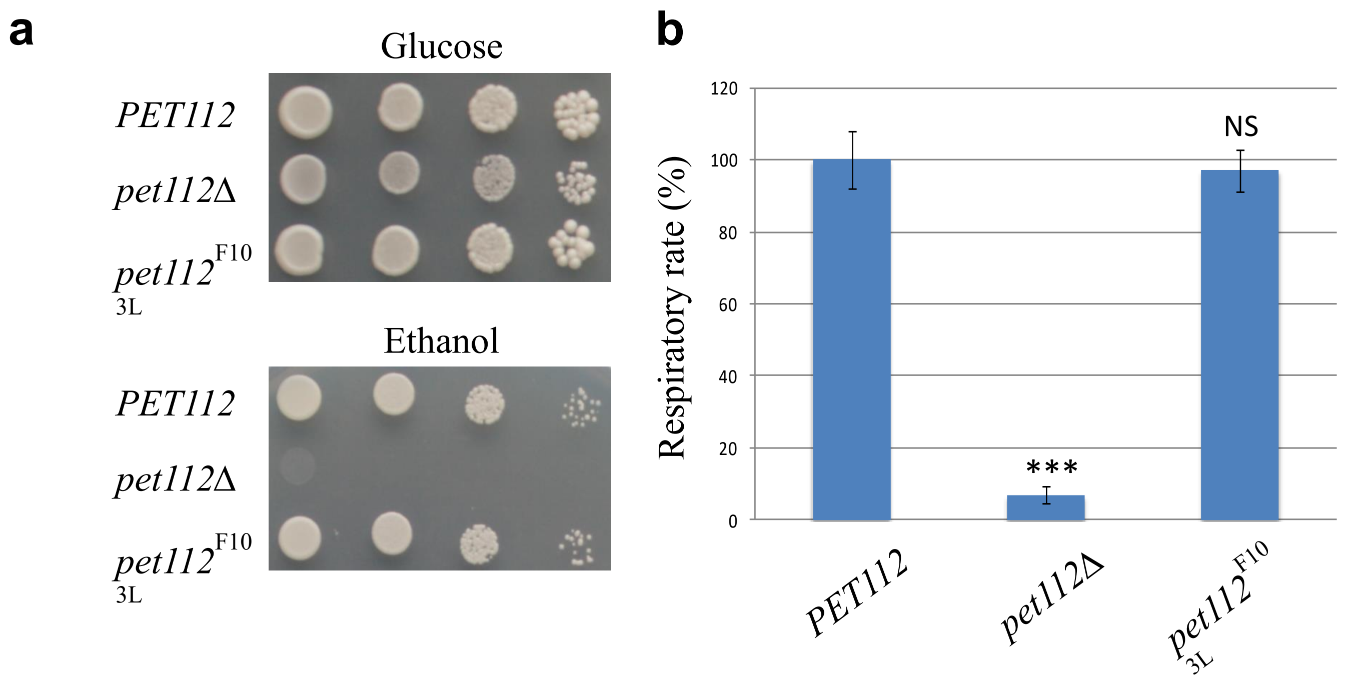

Legend: a) Oxidative growth: the *pet112Δ* strains harboring *PET112* wild-type allele, *pet112*^{F103L} mutant allele, or the empty vector were serially diluted and spotted on synthetic complete agar plates supplemented with 2% glucose or 2% ethanol and incubated at 28°C. (b) Oxygen consumption rate: cells were grown at 28°C in SC medium supplemented with 0.6% glucose. Values were normalized to the wild-type strain. The data are the results of at least three measurements, and the error bars indicate the standard deviation. Statistical analysis was performed by paired, two-tail Student's t test comparing mutant strains to wild type strain: ***p<0.001, NS = not significant.

Supplementary Figure 6: Oxygen consumption rate.

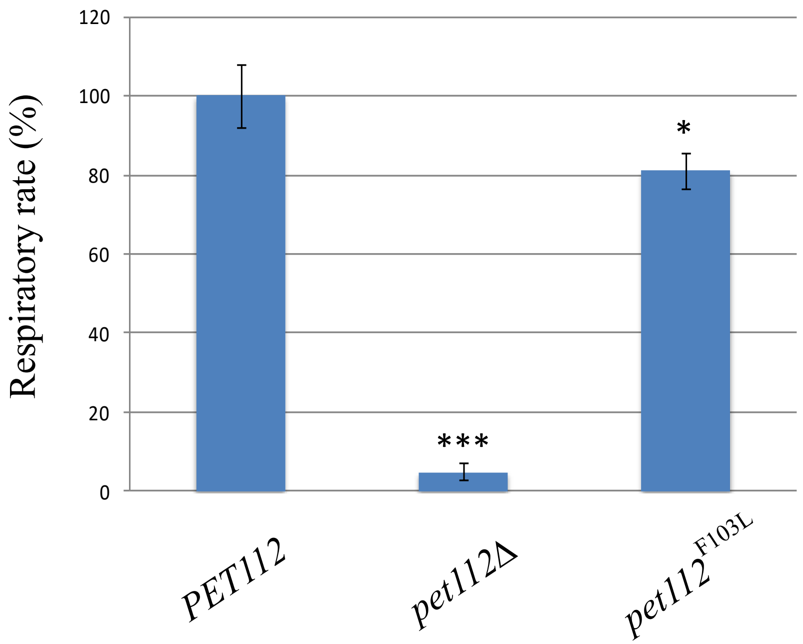

Legend: The *pet112Δ* strains harboring *PET112* wild-type allele, *pet112*^{F103L} mutant allele, or the empty vector were grown at 37°C in SC medium supplemented with 0.6% glucose. Values were normalized to the wild-type strain. The data are the results of at least three measurements, and the error bars indicate the standard deviation. Statistical analysis was performed by paired, two-tail Student's t test comparing mutant strains to wild type strain: ***$p<0.001$, *$p<0.05$.

Supplementary Figure 7: Uncropped images of Figure 3a

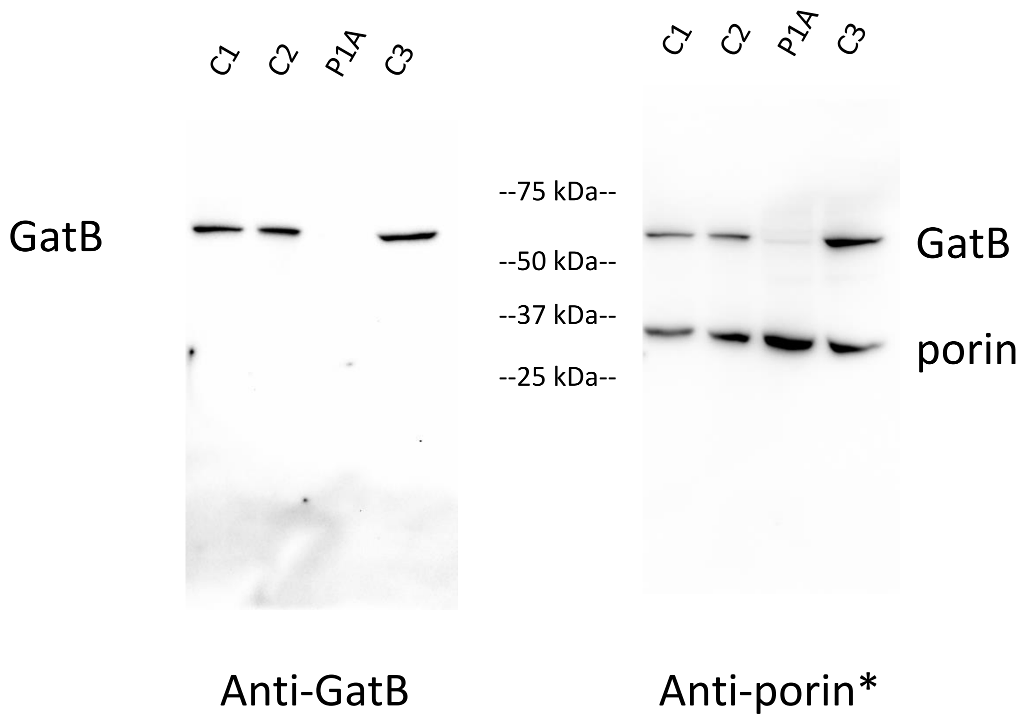

Legend: Uncropped images of Western blot shown in Figure 3 panel a with molecular weight markers. Please note that the same blot was used for both stainings: first by using an anti-GatB antibody (left image), thereafter the same blot (without stripping of anti-GatB and secondary antibodies) was incubated with anti-porin (right image)*. Therefore, on the right image there is some GatB signal present.

Supplementary Figure 8: Uncropped images of Figure 3b

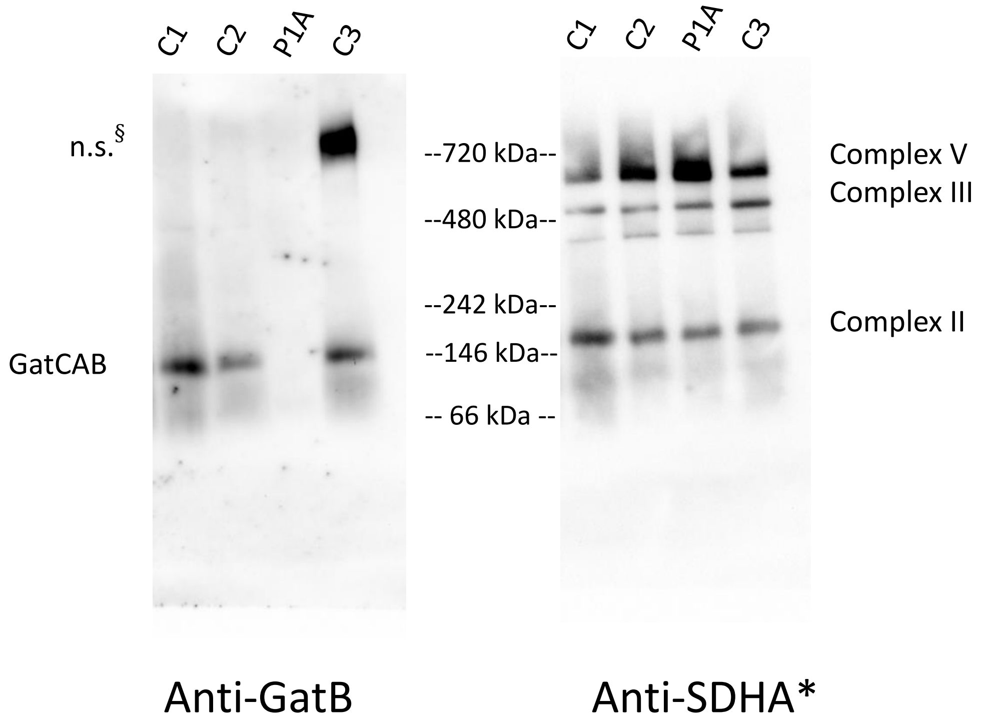

Legend: Uncropped images of Western blot shown in Figure 3 panel b with molecular weight markers. * Please note that the complex II blot (right image) was also stained with antibodies against complex III and complex V. § non-specific band only observed in control C3 but not in other controls.

Supplementary Figure 9: Uncropped images of Figure 3c

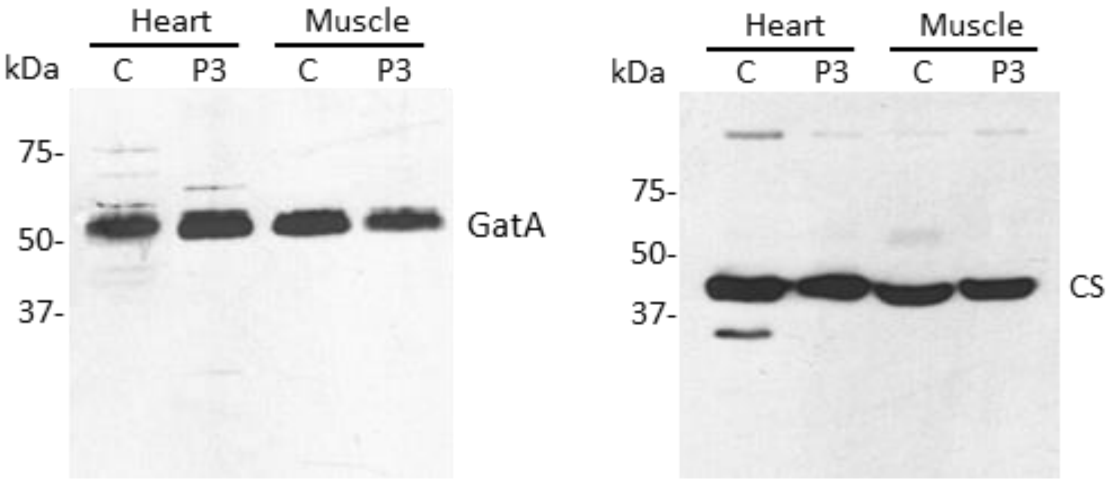

Legend: Uncropped images of Western blot shown in Figure 3 panel c with molecular weight markers.

Supplementary Figure 10: Uncropped images of Figure 3e

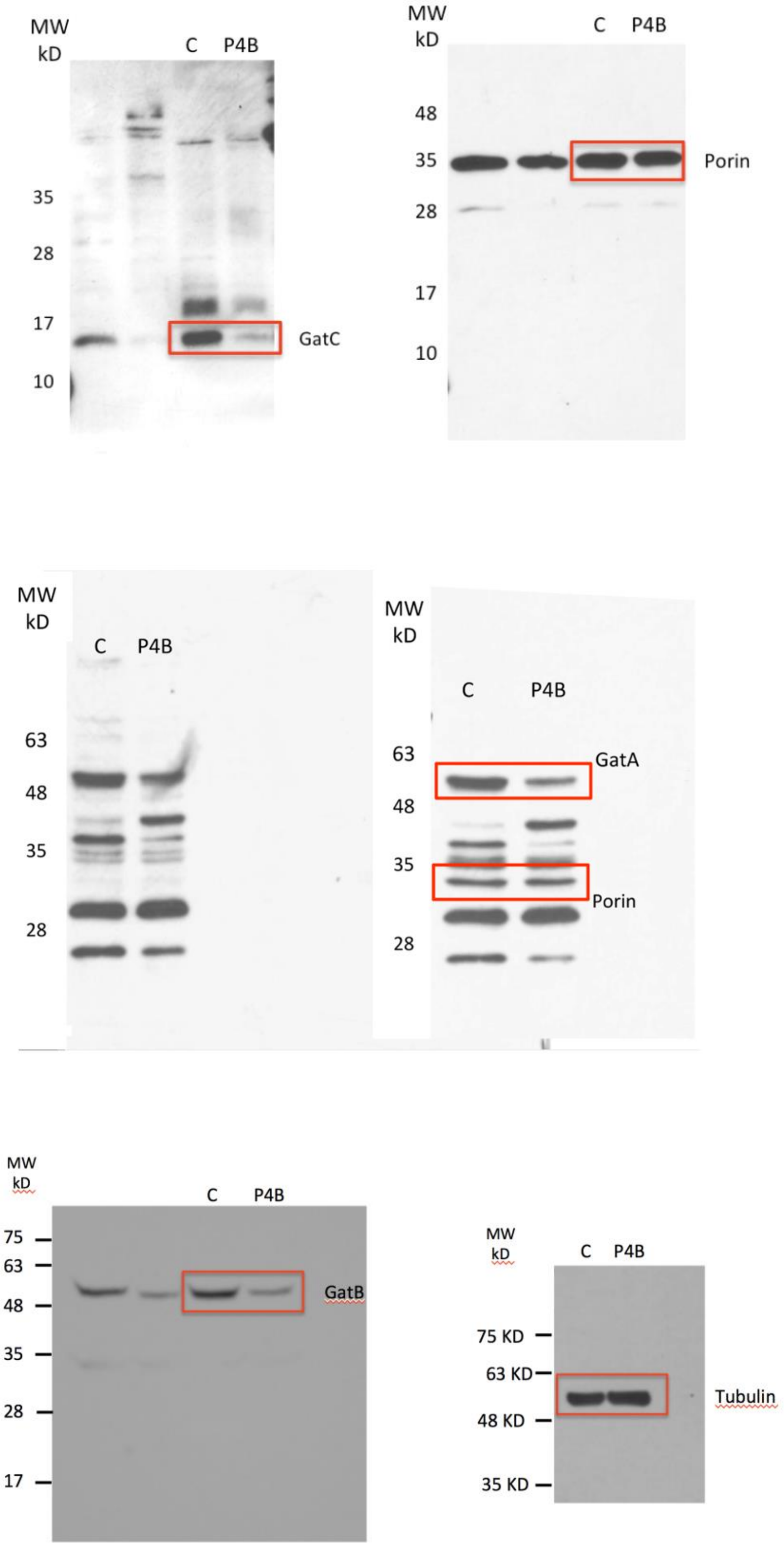

Legend: Uncropped images of Western blot shown in Figure 3 panel e with molecular weight marker indicated. The portion of the blots presented in Figure 3 panel e are indicated by a red box. Please, note that for GatA the same blot was used for both stainings: first by using an anti-GatA antibody (left image), thereafter the same blot (without stripping of anti-GatA and secondary antibodies) was incubated with anti-porin (right image). For GatB and GatC twin gels were carried out for problem and control antibodies.

Supplementary Figure 11: Uncropped images of Figure 3d

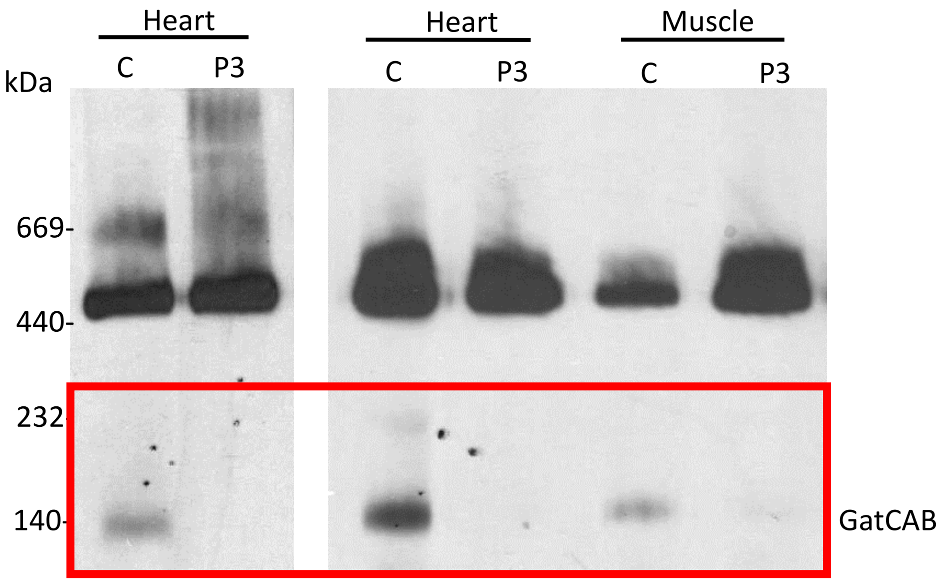

Legend: Uncropped images of Western blot shown in Figure 3 panel d with molecular weight markers. The portion of the blot from Figure 3 panel d presented in the paper is indicated by the red box. The band at 440 kDa was shown by 2D gel analysis not to be part of the GatCAB complex.

Supplementary Figure 12: Uncropped images of Figure 3f

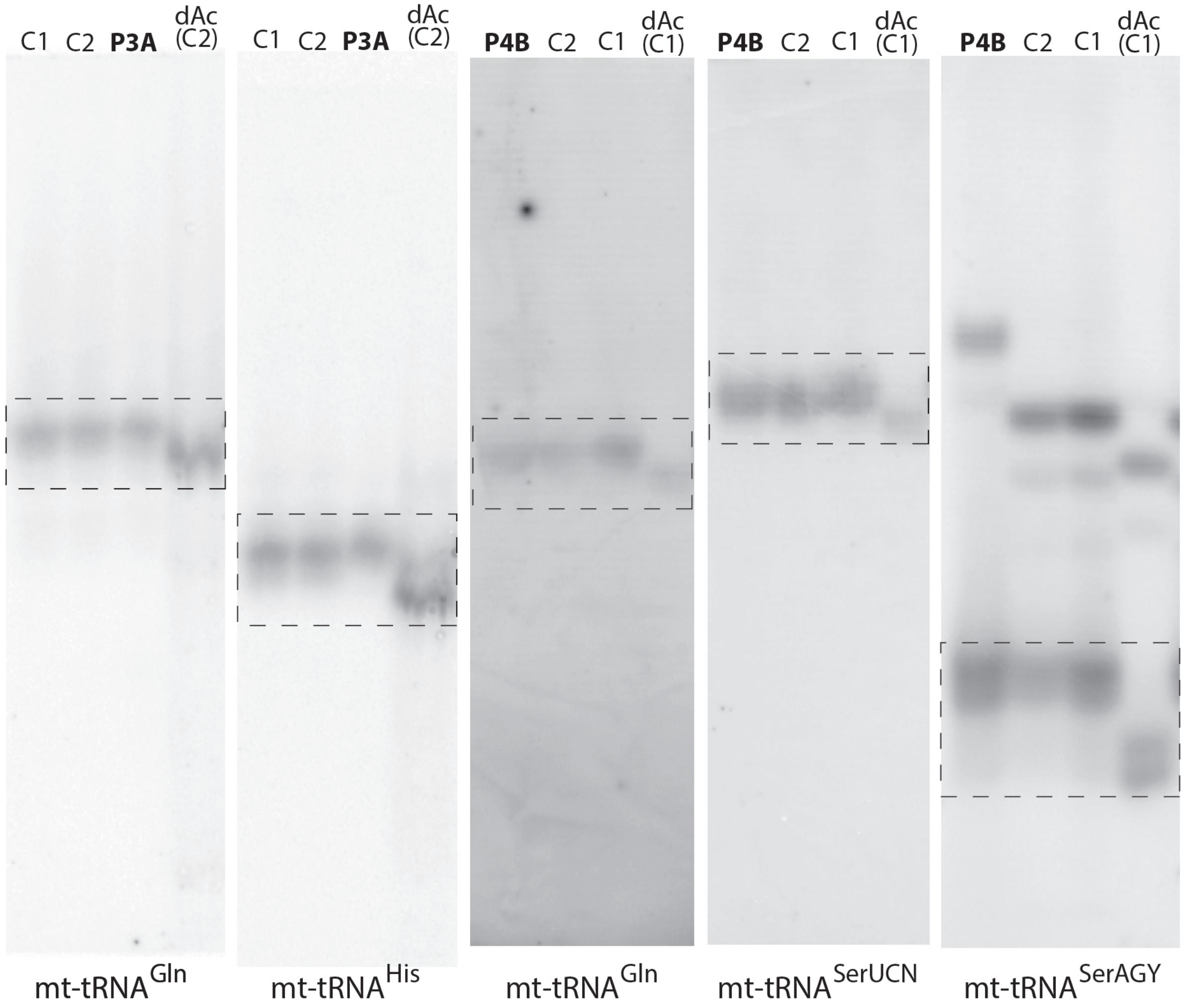

Legend: Uncropped images of northern blots shown in Figure 3 panel f. The portion of the blot from Figure 3 panel f presented in the main text is indicated by dashed lines.

Supplementary Figure 13: Uncropped images of Figure 3g

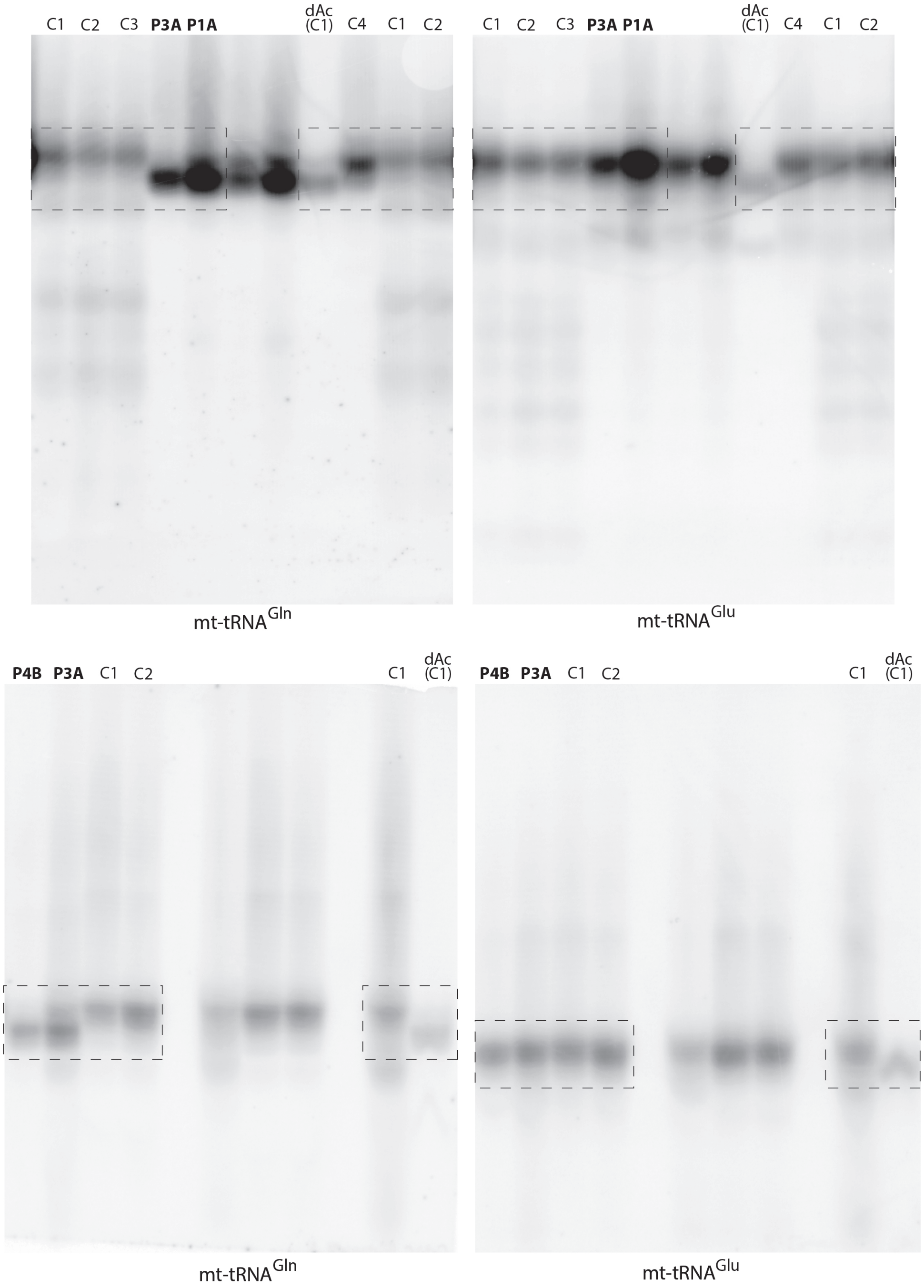

Legend: Uncropped images of northern blots shown in Figure 3 panel g. The portion of the blot from Figure 3 panel gpresented in the main text is indicated by dashed lines.

Supplementary Figure 14: Uncropped images of Figure 5
Panel a:

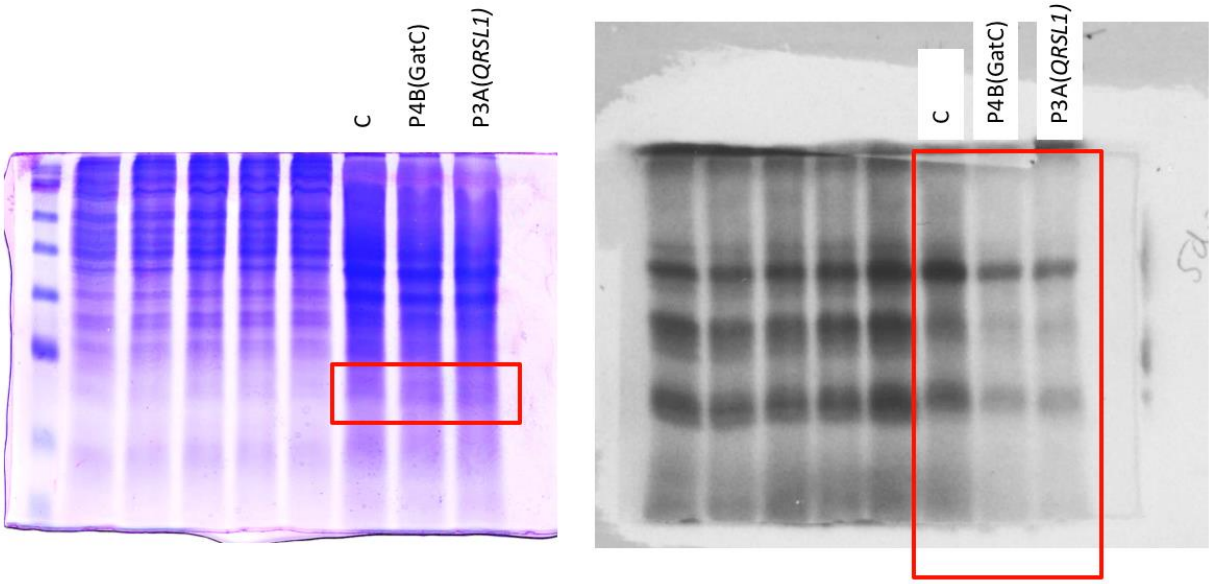

Panel b:

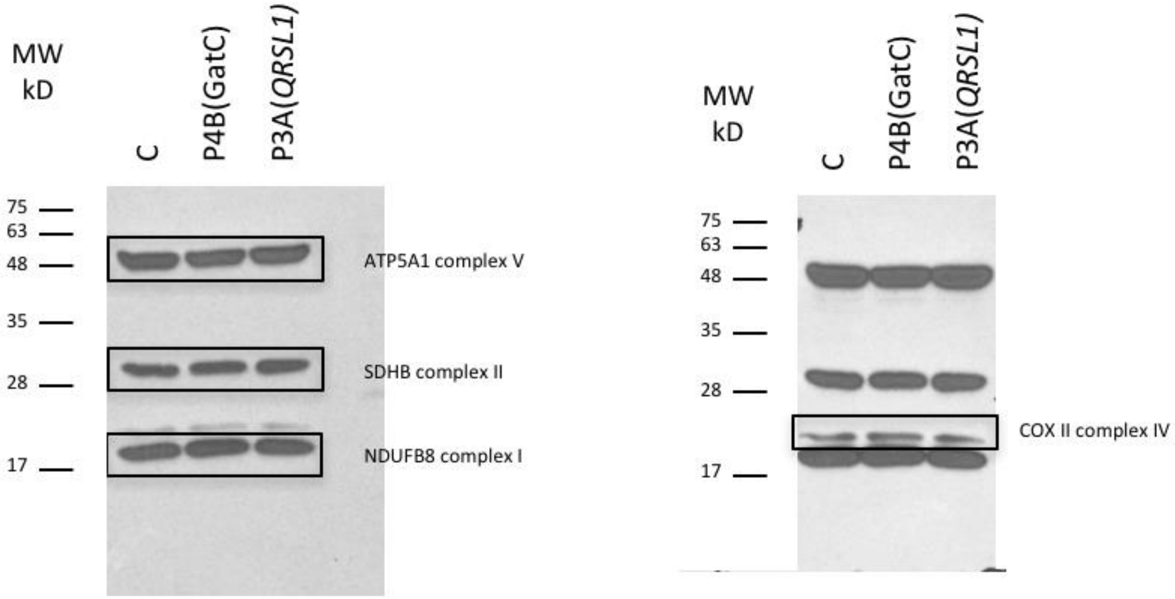

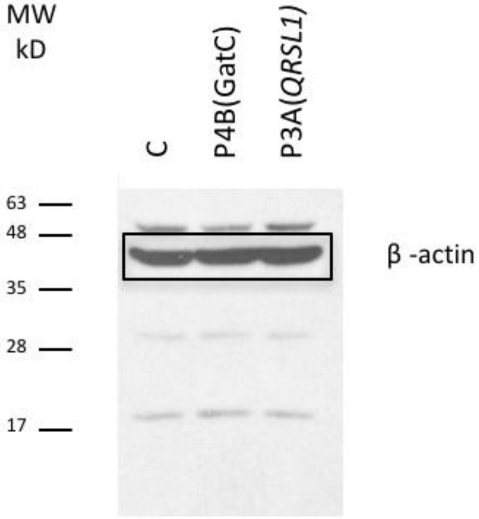
MW
kD
C
P4B(GatC)
P3A(*QRSL1*)
63
48
35
28
17
β -actin

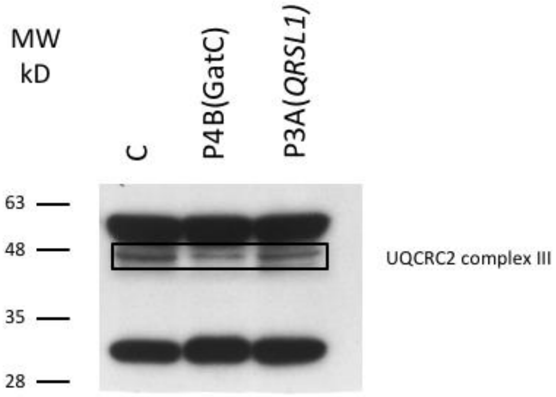
MW
kD
C
P4B(GatC)
P3A(*QRSL1*)
63
48
35
28
UQCRC2 complex III

Panel c:

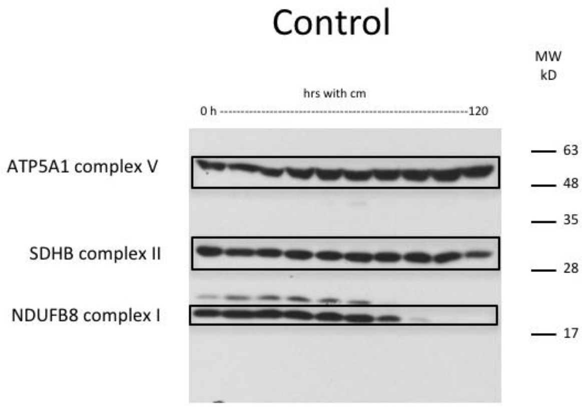
Control
MW
kD
hrs with cm
0 h --120
ATP5A1 complex V
SDHB complex II
NDUFB8 complex I
63
48
35
28
17

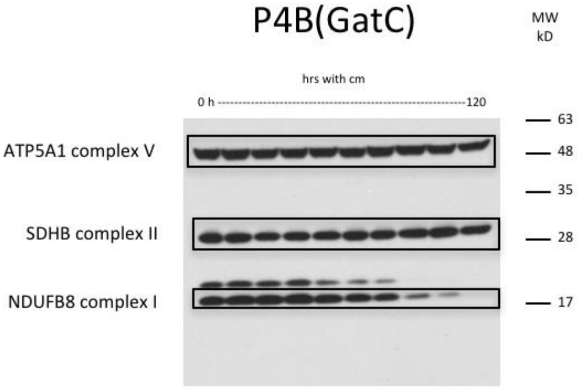
P4B(GatC)
MW
kD
hrs with cm
0 h --120
ATP5A1 complex V
SDHB complex II
NDUFB8 complex I
63
48
35
28
17

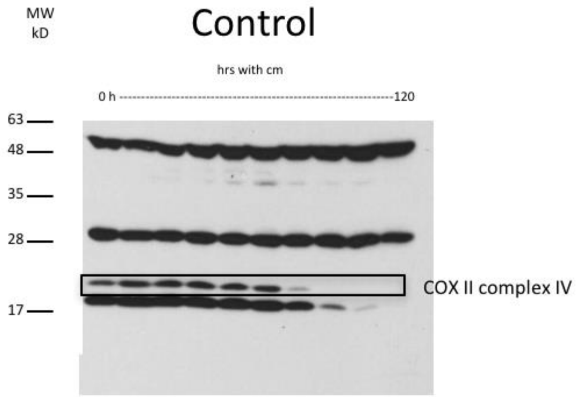
MW
kD
Control
hrs with cm
0 h --120
63
48
35
28
17
COX II complex IV

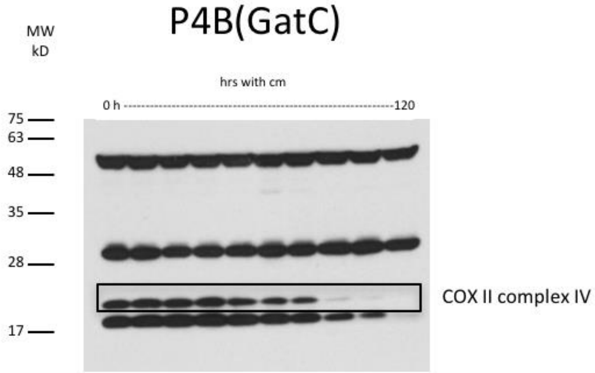
MW
kD
P4B(GatC)
hrs with cm
0 h --120
75
63
48
35
28
17
COX II complex IV

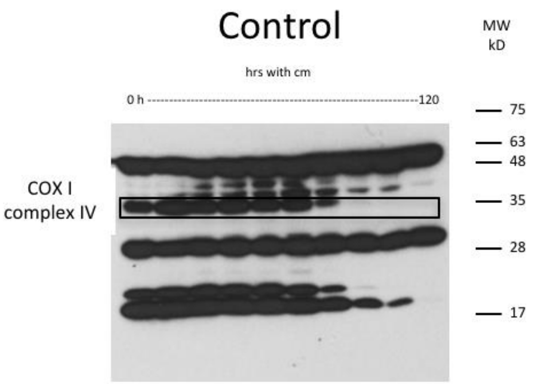
Control
MW
kD
hrs with cm
0 h
120
75
63
48
35
28
17
COX I
complex IV

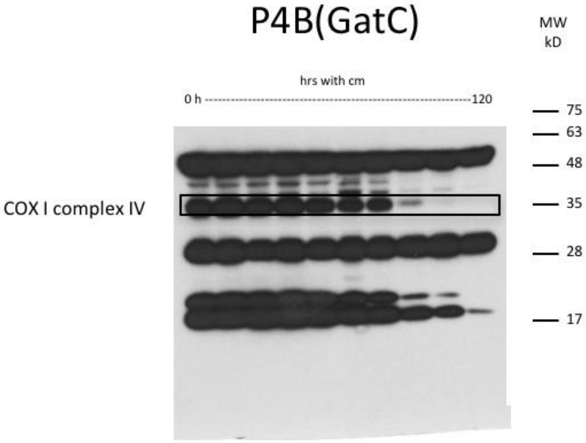
P4B(GatC)
MW
kD
hrs with cm
0 h
120
75
63
48
35
28
17
COX I complex IV

Panel d:

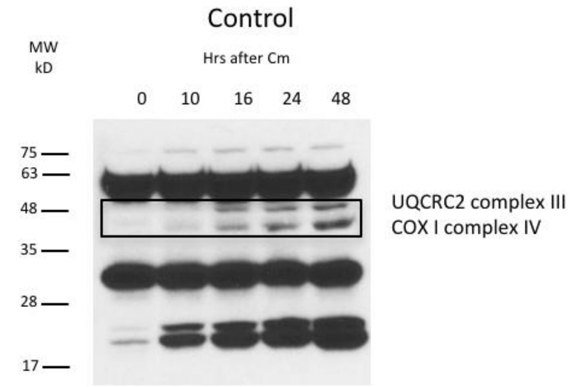
Control
MW
kD
Hrs after Cm
0 10 16 24 48
75
63
48
35
28
17
UQCRC2 complex III
COX I complex IV

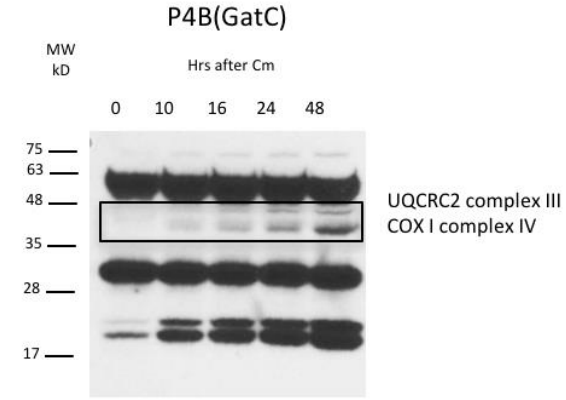
P4B(GatC)
MW
kD
Hrs after Cm
0 10 16 24 48
75
63
48
35
28
17
UQCRC2 complex III
COX I complex IV

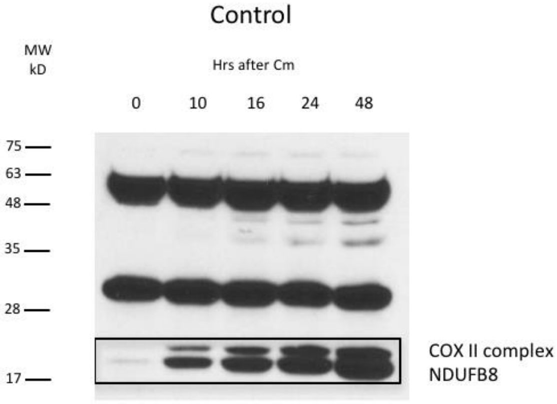
Control
MW
kD
Hrs after Cm
0 10 16 24 48
75
63
48
35
28
17
COX II complex
NDUFB8

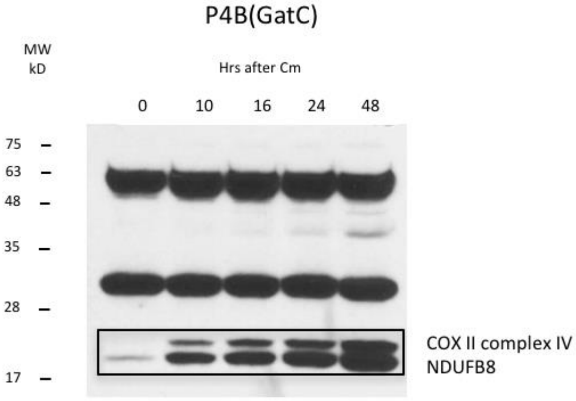
P4B(GatC)
MW
kD
Hrs after Cm
0 10 16 24 48
75
63
48
35
28
17
COX II complex IV
NDUFB8

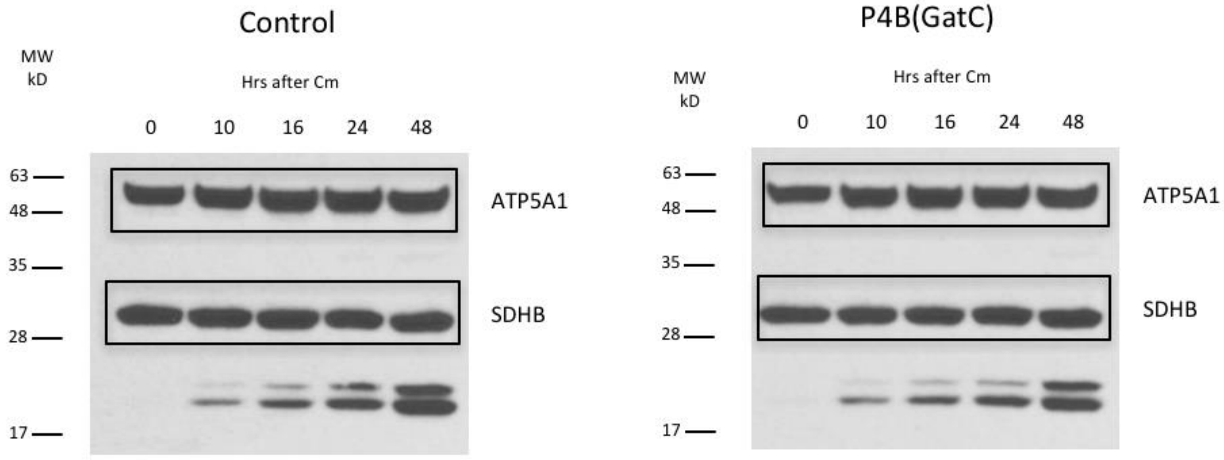

Panel e:

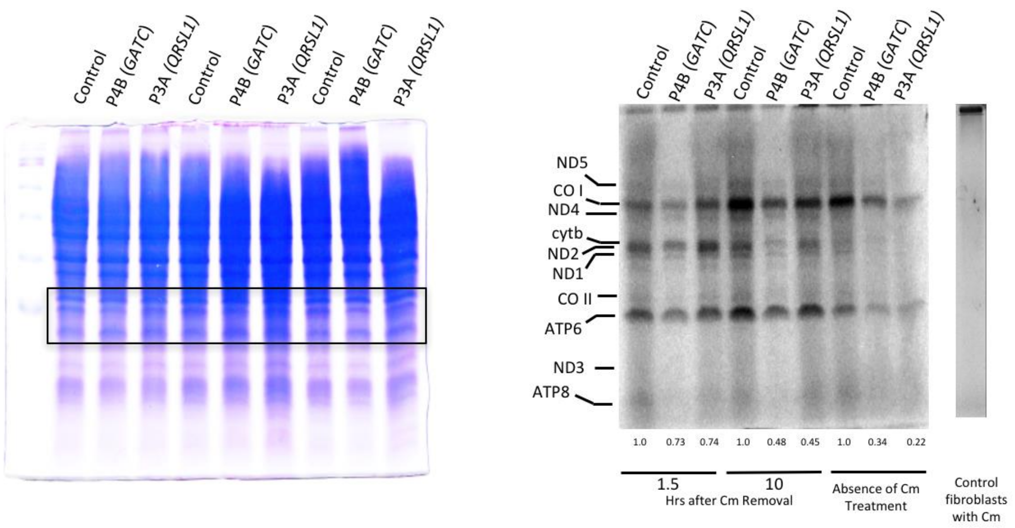

Legend: Uncropped images of mitochondrial labeled proteins and Western blot shown in Figure 5. In panel a and e images shown are uncropped and corresponding Coomassie blue staining is included with the region shown in the figure in a box. All images in Panel b but that for UQCRC2 complex III are from the same gel with different exposure times. They include molecular weight markers. In panels c and d control and P4B(GatC) images are shown in pairs treated in the same conditions and with the same exposure time.

Supplementary Figure 15: Uncropped images of Figure 6a

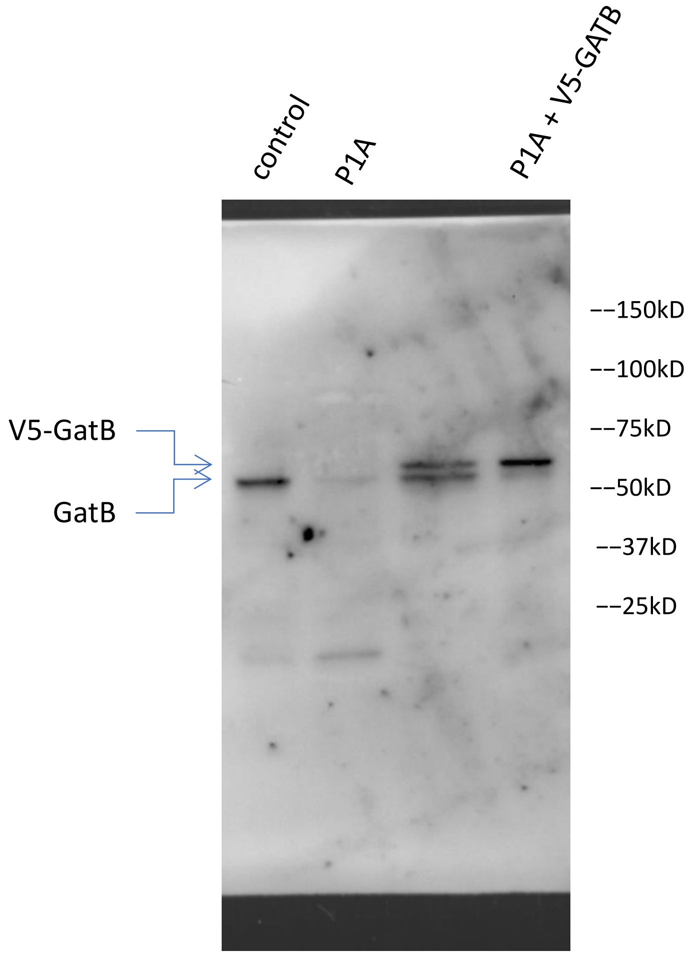

Legend: Uncropped images of Western blot shown in Figure 6 panel a with molecular weight markers

Supplementary Figure 16: Uncropped images of Supplementary Figure 3c

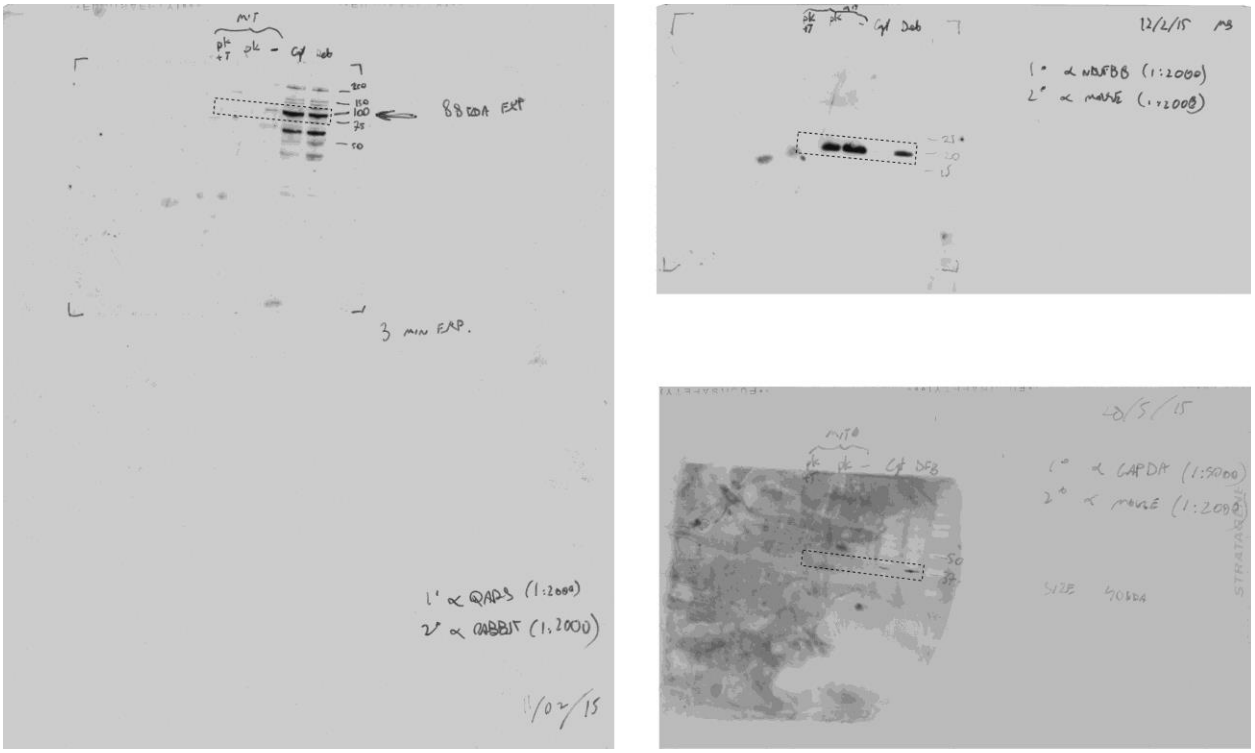

Legend: Uncropped images of Western blot shown in Supplementary Figure 3 panel c with molecular weight markers, date of experiments and antibody dilutions.

Supplementary Tables

Supplementary Table 1: Respiratory chain enzyme activities

Patient	Gene	Tissue	Complex I	Complex II	Complex III	Complex IV
**Ratio over citrate synthase**						
P1A	*GATB*	Fibroblasts	**73** (163–559)	440 (335–888)	**446** (570–1383)	**73** (288–954)
P1B	*GATB*	Fibroblasts	**117** (163–559)	621 (335–888)	667 (570–1383)	**204** (288–954)
P2A	*QRSL1*	Skeletal muscle	**2** (68-230)	186 (76-281)	**92** (182-1421)	**34** (228-1032)
		Liver	**44** (116-960)	2042 (660-4979)	**256** (1090-5570)	**36** (662-4220)
P3A	*QRSL1*	Heart muscle	**3** (144-376) -14.9SD	327 (235-356) 0.8SD	**12** (12-210) -1.7SD	**3** (11-32) -6.3 SD
		Skeletal muscle	**39** (98-271) -5.9 SD	298 (251-573) -0.8 SD	49 (19-172) 0.7 SD	5 (4-23) -2.5 SD
		Liver	**100** (162-730) -3.1 SD	2022 (2304-3311) -1.5 SD	**115** (128-315) -2.0 SD	9 (6-35) -2.0 SD
		Fibroblasts	192 (151-387) -0.5 SD	529 (471-919) -1.0 SD	26 (21-79) -0.9 SD	11 (6-25) 0.0 SD
P4B	*GATC*	Fibroblasts	277 (145-396) +0.4 SD	814 (297 – 863) +1.1 SD	86 (19 – 65) +1.7 SD	10 (6 – 23) -1.5 SD
			**I-III assay**	**Complex II**	**II-III assay**	**Complex IV**
P4A	*GATC*	Muscle	**6.3** (71-344)	226 (48-346)	**32** (69-294)	**84** (574-1846)
P5A	*GATC*	Muscle	**98** (131-444)	150 (80-190)	81 (40-140)	**533** (545-1256)

Legend: Respiratory chain enzyme activities listed as mU.U^{-1} citrate synthase, patient values provided followed by laboratory specific normal values in brackets, and for patients P3A and P4B standard deviations of normal distribution of log transformed values of controls. Reduced activities are listed in bold.

Supplementary Table 2: Results of exome sequencing

Patient P1A: *GATB*

Chr.	Genomic position [hg19]	Reference nucleotide	Variant nucleotide	Coverage	Variant reads	% variation	ExAC frequency	GoNL frequency	Gene	Isoform ID	cDNA change	Protein change	SIFT	PolyPhen-2	Mutation Taster
4	152640610	A	C	145	66	46%	0.0008%	n.a.	*GATB*	NM_004564	408T>G	Phe136Leu	Deleterious	Probably damaging	Disease causing
4	152638087	CT	-	92	36	39%	n.a.	0.10%	*GATB*	NM_004564	580_581del	Ser194Trpfs*15	n.a.	n.a.	n.a.
11	34969140	T	C	135	58	43%	0.0004%	n.a.	*PDHX*	NM_003477	329T>C	Leu110Ser	Deleterious	Probably damaging	Disease causing
22	42482262	C	T	81	33	41%	n.a.	n.a.	*NDUFA6*	NM_002490	390G>A	Met130Ile	Deleterious	Possibly damaging	Disease causing
8	66620160	G	C	52	17	33%	n.a.	n.a.	*MTFR1*	NM_014637	847G>C	Ala283Pro	Deleterious	Probably damaging	Disease causing

Patient P2A: *QRSL1*

Chr.	Genomic position [hg19]	Reference nucleotide	Variant nucleotide	Coverage	Variant reads	% variation	ExAC frequency	GoNL frequency	Gene	Isoform ID	cDNA change	Protein change	SIFT	PolyPhen-2	Mutation Taster
6	107096917	G	T	44	21	48%	n.a.	n.a.	*QRSL1*	NM_018292	398G>T	Gly133Val	Tolerated	Possibly damaging	Disease causing
6	107097074	C	A	114	52	46%	n.a.	n.a.	*QRSL1*	NM_018292	555C>A	Tyr185*	n.a.	n.a.	n.a.
2	44116923	C	T	91	44	48%	0.079%	n.a.	*LRPPRC*	NM_133259	4078G>A	Ala1360Thr	Deleterious	Probably damaging	Disease causing
6	155579035	G	A	82	36	44%	0.063%	n.a.	*TFB1M*	NM_016020	976C>T	Leu326Phe	Tolerated	Probably damaging	Disease causing
14	75368981	G	A	71	34	48%	0.0057%	n.a.	*DLST*	NM_001933	1310G>A	Arg437His	Deleterious	Probably damaging	Disease causing
17	18233950	G	A	107	50	47%	0.0037%	n.a.	*SHMT1*	NM_004169	1090C>T	Arg364Cys	Deleterious	Probably damaging	Disease causing
19	5705841	C	T	92	42	46%	0.31%	0.20%	*LONP1*	NM_004793	1309G>A	Val437Ile	Tolerated	Probably damaging	Disease causing
19	33424357	C	T	107	44	41%	0.0061%	n.a.	*CEP89*	NM_032816	886G>A	Val296Ile	Tolerated	Possibly damaging	Polymorphism

Patient P3A: *QRSL1*

Chr.	Genomic position [hg19]	Reference nucleotide	Variant nucleotide	Coverage	Variant reads	% variation	ExAC frequency	Gene	Isoform ID	cDNA change	Protein change	SIFT	PolyPhen-2	Mutation Taster
6	107100113	C	A	230	99	43%	0.0016%	*QRSL1*	NM_018292	587C>A	Thr196Asn	Deleterious	Probably damaging	Disease causing
6	107100116	G	A	237	100	42%	0.0016%	*QRSL1*	NM_018292	590G>A	Arg197Lys	Deleterious	Probably damaging	Disease causing
6	107100122	C	A	246	110	45%	0.0016%	*QRSL1*	NM_018292	596C>A	Pro199His	Deleterious	Probably damaging	Disease causing
6	107110973	G	T	170	92	54%	0.0041%	*QRSL1*	NM_018292	1279G>T	Ala427Leu	Deleterious	Probably damaging	Disease causing
6	107110974	C	T	173	92	53%	0.0041%	*QRSL1*	NM_018292	1280C>T				
1	2938410	A	C	166	157	95%	n.a.	*ACTRT2*	NM_080431	160A>C	Lys54Gln	Tolerated	Benign	Disease causing
1	16332653	G	A	106	53	50%	n.a.	*C1orf64*	NM_178840	322G>A	Ala108Thr	Tolerated	Benign	Polymorphism
1	228452027	C	T	107	52	49%	0.0017%	*OBSCN*	NM_052843	4796C>T	Ala1599Val	Tolerated	Possibly damaging	Polymorphism
1	228494271	G	A	201	109	54%	0.0034%	*OBSCN*	NM_052843	11858G>A	Arg3953His	Tolerated	Probably damaging	Polymorphism

1	228503805	T	C	113	49	43%	n.a.		*OBSCN*	NM_052843	13270T>C	Phe4424Leu	Deleterious	Benign	Polymorphism
5	149374878	TC	T	288	254	88%	n.a.		*TIGD6*	NM_030953	1033delG	Glu345fs	n.a.	n.a.	n.a.
10	95148899	T	A	57	33	58%	0.0494%		*MYOF*	NM_133337	1430A>T	Glu477Val	Deleterious	Possibly damaging	Disease causing
10	95191218	A	T	167	89	53%	0.3024%		*MYOF*	NM_133337	292T>A	Ser98Thr	Deleterious	Benign	Disease causing
11	46724728	A	C	35	35	100%	n.a.		*ZNF408*	NM_024741	587A>C	Glu196Ala	Tolerated	Benign	Polymorphism
16	4625656	G	A	84	33	39%	0.0111%		*C16orf96*	NM_001145011	1175G>A	Arg392His	Tolerated	Probably damaging	Polymorphism
16	4626370	G	A	100	65	65%	0.0379%		*C16orf96*	NM_001145011	1889G>A	Arg630Gln	Tolerated	Possibly damaging	Polymorphism
16	10524659	C	G	157	147	94%	n.a.		*ATF7IP2*	NM_001256160	182C>G	Thr61Arg	Tolerated	Benign	Polymorphism
6	67314182	T	C	79	32	41%	0.0108%		*PLEKHG4*	NM_001129727	235T>C	Ser79Pro	Tolerated	Benign	Polymorphism
16	67319257	A	G	162	71	44%	0.3916%		*PLEKHG4*	NM_001129727	2260A>G	Thr754Ala	Tolerated	Probably damaging	Polymorphism
18	47405425	T	G	204	187	92%	0.0008%		*MYO5B*	NM_001080467	3166A>C	Met1056Leu	Tolerated	Benign	Disease causing
19	48305564	T	A	19	4	21%	n.a.		*TPRX1*	NM_198479	704A>T	Asn235Ile	Deleterious	Benign	Polymorphism
19	48305646	G	A	16	5	31%	0.0001%		*TPRX1*	NM_198479	622C>T	Pro208Ser	Deleterious	Benign	Polymorphism
**Patient 4B: *GATC***															
**Chr.**	**Genomic position [hg19]**	**Reference nucleotide**	**Variant nucleotide**	**Coverage**	**Variant reads**	**% variation**	**ExAC frequency**	**GME frequency**	**Gene**	**Isoform ID**	**cDNA change**	**Protein change**	**SIFT**	**PolyPhen-2**	**Mutation Taster**
12	120884611	T	G	99	99	100%	n.a.	n.a.	*GATC*	NM_176818	233T>G	Met78Arg	Deleterious	Probably damaging	Disease causing
1	223722710	T	C	129	129	100%	n.a.	0.12%	*CAPN8*	NM_001143962	1408A>G	Thr470Ala	Deleterious	Possibly damaging	Disease causing
1	228476575	C	T	29	29	100%	n.a.	n.a.	*OBSCN*	NM_001271223	11612C>T	Thr3871Ile	Tolerated	Benign	Polymorphism
1	240370222	G	A	115	113	98%	0.0016%	0.05%	*FMN2*	NM_001305424	2122G>A	Val708Met	Deleterious	Benign	Polymorphism
1	44422087	A	G	107	105	98%	n.a.	n.a.	*IPO13*	NM_014652	917A>G	His306Arg	Tolerated	Benign	Disease causing
2	11284184	T	C	116	115	99%	0.2859%	0.25%	*C2orf50*	NM_182500	436T>C	Phe146Leu	Tolerated	Benign	Disease causing
4	169812138	A	G	143	141	98%	n.a.	n.a.	*PALLD*	NM_001166108	2030A>G	Lys677Arg	Deleterious	Probably damaging	Disease causing
7	103180847	T	C	81	81	100%	0.0008%	n.a.	*RELN*	NM_005045	6727A>G	Ser2243Gly	Deleterious	Probably damaging	Disease causing
11	18159591	G	A	57	56	98%	0.0033%	n.a.	*MRGPRX3*	NM_054031	842G>A	Arg281His	Tolerated	Benign	Polymorphism
11	18956216	A	T	123	123	100%	0.0024%	n.a.	*MRGPRX1*	NM_147199	116T>A	Leu39His	Deleterious	Probably damaging	Polymorphism
15	77321913	C	G	153	152	99%	n.a.	n.a.	*PSTPIP1*	NM_003978	560C>G	Ala187Gly	Deleterious	Possibly damaging	Disease causing
19	56539847	G	A	11	11	100%	0.1574%	0.35%	*NLRP5*	NM_153447	2248G>A	Ala750Thr	Tolerated	Benign	Polymorphism
19	58945021	T	C	106	106	100%	n.a.	n.a.	*ZNF132*	NM_003433	1789A>G	Lys597Glu	Tolerated	Benign	Polymorphism

Abbreviations: Chr. = Chromosome; SNP ID = dbSNP138 identifier; SNP frequency according to Exome Aggregation Consortium (ExAC) database, GoNL database, Greater Middle East (GME) Variome.

Supplementary Table 3: Antibody sources

Antigen	Source	Reference	Lab	Subjects	Dilution
GatA (QRSL1)	Abcam	ab83842	Denver	P3	1:1000
	Thermo-Fisher	PA5-48981	Spain	P4	1:500
GatB (PET112)	Sigma	HPA042610	Nijmegen	P1,2	1:1000
	Abcam	Ab55606	Madrid	P4	1:1000
GatC	Lifespan biosciences	LS-C410140	Madrid	P4	1:1000
Citrate synthase	Abcam	Ab0129095	Denver	P3	1:2000
V5	Invitrogen	R960-25	Nijmegen	P1,2	1:5000
MTCOXI	Abcam	ab14705	Madrid	P4	1:1000
Total OXPHOS human	Abcam	ab110411	Madrid	P4	1:1000
VDAC1	Abcam	ab154856	Madrid	P4	1:3000
HSP60	Abcam	ab46798	Nijmegen	P1	1:1000
QARS	GeneTex	GTX108713	Cambridge	NA	1:1000
NDUFB8	Abcam	ab110242	Cambridge	NA	1:2000
GAPDH	Abcam	ab9482	Cambridge	NA	1:500
NDUFS2	Abcam	ab110249	Denver	P3	1:2000

Legend: Antibodies for Western blotting were obtained from the listed sources. NA = not applicable

Supplementary Table 4: ClinVar submission

Gene	Mutation	ClinVar accession
GATB	NM_004564.2:c.580_581del	SCV000787745
GATB	NM_004564.2:c.408T>G	SCV000787746
QRSL1	NM_018292.4:c.555C>A	SCV000787747
QRSL1	NM_018292.4:c.398G>T	SCV000787748
QRSL1	NM_018292.4:c.587_596delinsACAAAAATCA	SCV000787749
QRSL1	NM_018292.4:c.1279_1280delinsTT	SCV000787750
GATC	NM_176818.2:c.233T>G	SCV000787751

Legend: The table provides the ClinVar accession numbers. ClinVar can be accessed at: http://www.ncbi.nlm.nih.gov/clinvar/
